# Recent Advances in Pharmaceutical and Medical Applications in the Area of Selected Porphyrinoids Connected with PLGA or PLGA-Based Modalities

**DOI:** 10.3390/polym17233190

**Published:** 2025-11-29

**Authors:** Patrycja Koza, Jakub Kubiak, Tomasz Goslinski, Tomasz Koczorowski

**Affiliations:** 1 Poznan University of Medical Sciences, Chair and Department of Chemical Technology of Drugs, Rokietnicka 3, 60-806 Poznan, Poland; patrycja.koza@student.ump.edu.pl (P.K.); 86216@student.ump.edu.pl (J.K.); 2 Poznan University of Medical Sciences, Doctoral School, Bukowska 70, 60-812 Poznan, Poland

**Keywords:** photodynamic therapy, photosensitizers, PLGA, polymers, porphyrins

## Abstract

The challenges associated with solubility and bioavailability of porphyrinoid-type photosensitizers in photodynamic therapy require solutions that are based on modern drug carriers, including polymeric nanoparticles. With that in mind this review discusses poly(lactic-*co*-glycolic acid, PLGA)-based polymeric nanoparticles encapsulating selected well-known photosensitizers, such as protoporphyrin IX, tetrahydroxyphenylporphyrin, chlorin e6, and tetracarboxyphenylporphyrin, with a view to the physicochemical and biological properties. Also discussed are their potential medical applications towards photodynamic and sonodynamic therapy. PLGA-based nanoparticles, encapsulating photosensitizers, were analysed in terms of particle size, surface charge, morphology, loading efficiency, release kinetics, and stability. Moreover, the cellular uptake and subcellular localisation of carriers were considered in correlation to polymer composition and surface functionalisation. Special attention was given to how PEGylation, lipid-hybrid coatings, or the incorporation of additional therapeutic or imaging agents has modulated both the physicochemical properties and biological activities of photosensitizers. The comparative assessment of different porphyrinoid-based photosensitizers highlighted how hydrophobicity, amphiphilicity, and molecular structure have an influence on encapsulation efficiency and therapeutic outcomes. Furthermore, issues such as the premature release of photosensitizers, along with limited bioavailability, and limited penetration through biological barriers were addressed as well as some proposed mitigation strategies. Overall, this review highlights the versatility of PLGA nanoparticles as a powerful platform for photosensitizer delivery, with promising implications for advancing polymer-based nanomedicine and improving the efficacy of photodynamic therapy.

## 1. Introduction

**Figure 1:**
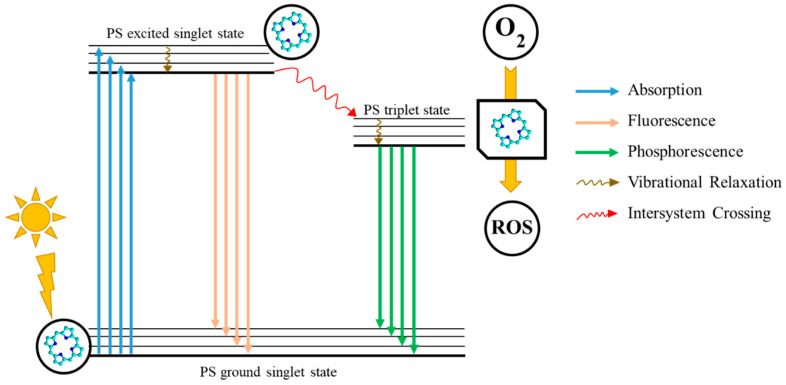
A schematic representation of the photodynamic reaction, in a nutshell, constituting the foundation of photodynamic therapy; PS—photosensitizer; ROS—Reactive Oxygen Species.

Photosensitizers (PSs) constitute a vital class of therapeutic agents used in combination with nanocarriers to tackle both severe and benign medical issues. They are highly important for photodynamic therapy (PDT), which is a minimally invasive anticancer or antimicrobial treatment that involves a PS, visible light, and molecular oxygen to generate reactive oxygen species (ROS), which are capable of selectively destroying cancer cells or pathogens (Figure 1) [1]. Upon light activation, PS undergoes transitions to an excited state initiating photochemical reactions, which then lead to cytotoxic ROS, particularly singlet oxygen as the most active agent [2]. PDT demonstrates highly localised action, resulting in minimal damage to surrounding healthy tissues—a significant improvement over systemic chemotherapies [3,4]. However, PDT efficacy is restricted by its limited tissue light penetration (typically <1.5 cm) and the pharmacokinetics of PS [5]. Due to its precision and tissue-sparing properties, modern applications of PDT include the treatment of various cancers, such as those of the skin, bladder, oesophagus, lung, and pancreas [1,6].

Since the early 20th century, the discovery of PDT, and the development of the first clinically approved PS (Photofrin^®^) in the 1970s, significant advancements have focused on optimising those properties of photosensitizers which make them so effective. Ideally photosensitizers should be water-soluble, non-toxic in the dark, biocompatible, and capable of producing sufficient ROS upon irradiation [7]. Among PSs’ classes, porphyrins and their derivatives, including protoporphyrin IX (PPIX), have been widely studied due to their photostability, ROS generation efficiency, and their ability to accumulate in tumours. Nevertheless, their clinical use is limited due to poor solubility and suboptimal absorption in the therapeutic window (600–800 nm) [8]. To address these issues, various strategies have been employed, such as structural modification, conjugation with bioactive molecules, or encapsulation in drug delivery systems [9,10]. Nanocarriers, in particular, enhance the solubility of PS, improve tissue targeting, and enable controlled release, ultimately improving PDT outcomes.

**Figure 2:**
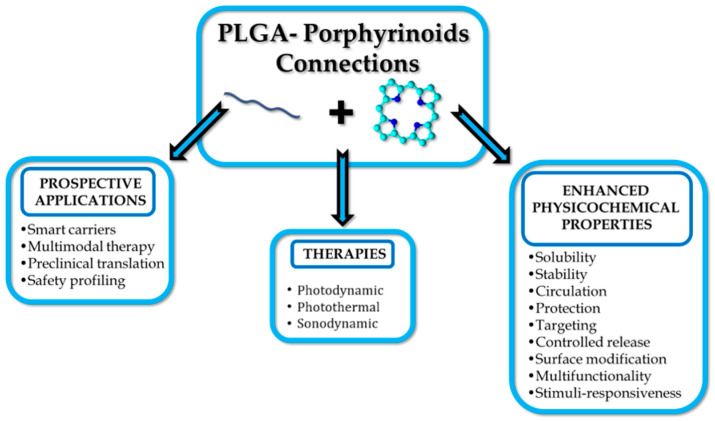
PLGA or PLGA-based carriers with porphyrinoids address the challenges of therapy.

Polymers have become indispensable in medicine because of their tunable properties, biocompatibility, and structural versatility. They are widely used in drug delivery, tissue engineering, and medical devices, offering advantages such as controlled release, targeted delivery, and enhanced bioavailability (Figure 2) [11]. Commonly utilised materials include polyethylene glycol (PEG), polylactic acid (PLA), and poly(lactic-*co*-glycolic acid) (PLGA), all of which support stimuli-responsive mechanisms to optimise therapeutic efficacy while minimising systemic toxicity [12,13,14]. Polymer-based delivery platforms have found applications across diverse medical fields, including cancer therapy, immunisation, and inflammation control [13]. That said, these systems also present certain limitations and challenges, such as immunogenic responses and variable rates of degradation. Research continues to refine polymer chemistry and nanofabrication techniques to mitigate those issues [11].

One of the most important applications of polymer-based drug delivery systems (DDSs) is their potential use in the preparation of pharmaceutical formulations for cancer therapy. DDSs offer significant advantages, overcoming the constraints of conventional chemotherapy. Many anticancer drugs have limited water solubility and exhibit broad systemic distribution, which can lead to dose-limiting toxicities. Polymeric nanocarriers, such as nanoparticles (NPs), including micelles and dendrimers, can enhance the solubility of anticancer drugs, prolong their circulation time, and improve targeting to pathological tissues [15,16,17,18]. Polymer-based DDSs can be employed for the encapsulation of diverse therapeutic agents, including hydrophobic small molecules, proteins, and nucleic acids, while protecting them from degradation and enabling their release in response to environmental triggers, such as pH or redox changes [15].

**Figure 3:**
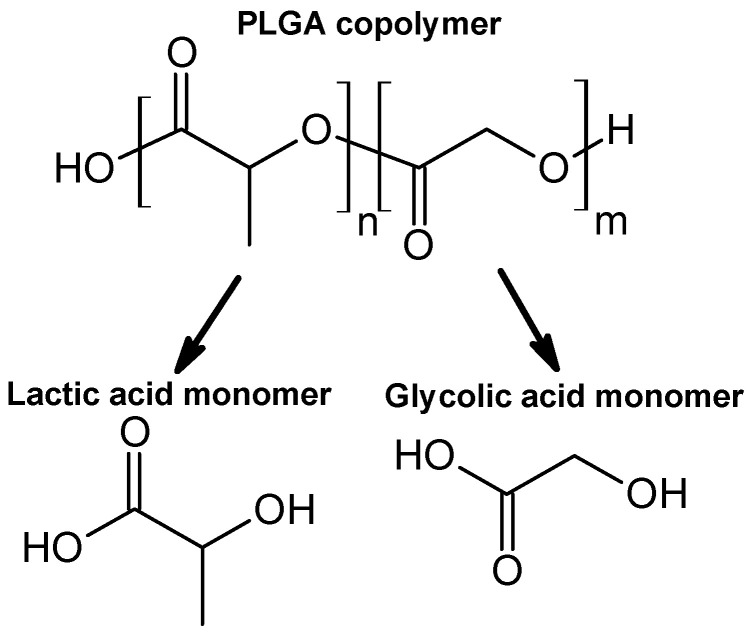
The chemical structure of PLGA copolymer and its monomers.

Upon evaluation of PDT strategies, when considering polymeric drug delivery systems, PLGA (Figure 3) emerges as one of the most promising materials for encapsulating and delivering photosensitizers [19]. As an FDA-approved, biodegradable copolymer consisting of lactic and glycolic acid monomers at different ratios, e.g., 75:25 or 50:50, PLGA offers controllable degradation profiles, high biocompatibility, and the aforementioned encapsulation of both hydrophilic and hydrophobic molecules [20]. Functionalization techniques, such as PEGylation or ligand conjugation, further enhance its hydrophilicity, targeting capacity, and drug loading efficiency [21]. PLGA-based formulations facilitate extended drug release, site-specific delivery, and modulation of the immune system. Notably, PLGA nanoparticles (PLGA NPs) tend to accumulate in the liver and spleen upon intravenous administration, whereas subcutaneous or intranodal routes can facilitate lymphatic targeting, particularly valuable in cancer immunotherapy [22].

In this review, we present the specific aspects of the last 20 years of research on PLGA-based nanoparticles encapsulating selected porphyrinoid-type photosensitizers from porphyrin and chlorin subclasses, such as protoporphyrin IX, tetrahydroxyphenylporphyrin, chlorin e6, and tetracarboxyphenylporphyrin. We intentionally excluded phthalocyanines (Pcs) as photosensitizers from this review due to an already sufficient amount of data regarding PLGA-porphyrin nanosystems. We do however acknowledge that Pcs encapsulated in biodegradable PLGA nanoparticles combine the strong near-IR absorption and high triplet-state yields of Pcs with the biocompatibility, colloidal stability and controlled-release behaviour of PLGA carriers, thus improving aqueous solubility, reducing Pc aggregation, and enhancing tumour accumulation and photodynamic efficacy in vitro and in vivo [23,24,25]. However, to date, several reviews regarding phthalocyanines have already been published, which sufficiently highlight the advantage of PLGA-based carriers in overcoming the aforementioned limitations of Pcs as photosensitizers in PDT [26,27].

As is outlined in the subchapters below, we analysed to what extent encapsulation of photosensitizers in PLGA or PLGA-based carriers alters the physicochemical properties and/or enhances the efficacy towards PDT of selected porphyrinoid photosensitizers. In this context, the unique potential of PLGA in addressing current challenges in PDT and advancing the field of polymer-based nanomedicine is demonstrated.

## 2. Selected Porphyrinoids in Connection with PLGA

### 2.1. Protoporphyrin IX in Polymeric Nanoparticles

Protoporphyrin IX (PPIX) is one of the most widely explored photosensitizers. PPIX is a key intermediate in the heme biosynthesis pathway, playing a crucial role in cellular metabolism and oxygen transport [28]. It was first identified in the early 20th century by German chemist Hans Fischer, who extensively studied porphyrins and was awarded the Nobel Prize in 1930 for his work on heme and chlorophyll [29]. As a highly conjugated, fluorescent molecule, PPIX has served as a natural photosensitizer with applications in PDT and medical imaging. Its accumulation in cells, influenced by enzymatic activity and metabolic regulation, has been widely studied for diagnostic and therapeutic purposes, particularly in cancer research. Understanding the biochemical properties and functional significance of PPIX provides valuable insights into its potential biomedical applications and its role in various pathological conditions [29].

While PPIX is a promising photosensitizer due to its tumour selectivity and photodynamic efficiency, its clinical use is limited by several major challenges. The first one is low water solubility, followed by a tendency to aggregate in aqueous solutions, which complicates administration and, thus, reduces its ability to generate singlet oxygen [30]. Moreover, PPIX activation wavelength (approx. 630 nm) limits deeper tissue penetration, reducing efficacy in deep-seated tumours [9]. Its rapid metabolism by ferrochelatase decreases its intracellular accumulation, while incomplete tumour selectivity may lead to damage in surrounding healthy tissues [31]. Additionally, PPIX undergoes photobleaching upon prolonged exposure to light, thereby diminishing its therapeutic effect. Last but not least, patient responses vary due to differences in metabolic enzyme activity, and side effects such as inflammation, pain, and prolonged photosensitivity may occur. These challenges severely constrain PPIX’s clinical use and highlight the need for optimised drug delivery, combination therapies, and improved light administration techniques to enhance PDT outcomes [32]. To address the first-mentioned challenge, numerous studies have been conducted on encapsulating PPIX, or its prometabolite, 5-aminolevulinic acid, into PLGA nanoparticles. In some nanoformulations, additional nanoparticles have been incorporated to enhance photodynamic or imaging outcomes.

#### 2.1.1. Protoporphyrin IX in PLGA Nanoparticles

One of the largest contributions in formulating PPIX/PLGA nanocarriers was made by da Silva and co-workers, who developed a nanoparticulate polymeric carrier system to deliver PPIX in its active, non-aggregated form to the viable layers of the skin, thus improving the effectiveness of PDT for skin cancers [33]. The nanoparticles were prepared using the nanoprecipitation method with Pluronic P-127 as a surfactant, with a high encapsulation efficiency of 67.7%. The resulting nanocarriers revealed a drug content of 50.3 µg/mg, an average size of 290 nm, and a zeta potential of −32.3 mV. Interestingly, while the blank nanoparticles initially measured 176.6 nm in diameter, the addition of PPIX resulted in a significant size increase of approximately 64%, indicating the successful incorporation of the photosensitizer. These formulation parameters were critical, as they directly influenced the penetration and retention of PPIX in the skin, which was subsequently analysed in the biological studies. The system’s potential for enhancing PPIX penetration and retention in the skin was evaluated by the researchers in a series of biological studies. In vitro release experiments demonstrated that nanoparticles significantly improved PPIX retention in both the stratum corneum (SC) and the epidermis plus dermis ([EP+D]) compared to a control solution. Retention levels were approximately 23 times higher in the SC and 10 times higher in [EP+D] when nanoparticles were used. For in vivo studies, the team applied PPIX-loaded nanoparticles or a PEG 300 control solution to the dorsum of healthy hairless mice for 24 h. Skin samples were removed post-euthanasia, and PPIX content was quantitatively assessed. The results showed that nanoparticles enhanced drug retention by a factor of 2.0 in the SC and 3.0 in [EP+D], the latter being the target site for topical PDT. Taking into account the PPIX lipophilic nature, which inherently limits its skin penetration, the results showed that the prepared nanoparticulate delivery system significantly improved both in vitro and in vivo PPIX retention and penetration through the skin, offering a promising avenue for more efficient and targeted photodynamic therapy for skin conditions [33]. Taken together, these findings confirmed that the nanoparticle system provided a practical way to overcome PPIX’s intrinsic lipophilicity, thereby enhancing its therapeutic potential for skin-targeted PDT.

Later, da Silva and co-workers extended their studies on PPIX/PLGA nanoparticles by in vitro release tests [34]. The assessment conducted in a Franz-type diffusion cell system demonstrated a controlled release profile. Within the first two hours, about 37.0% of the encapsulated PPIX was released, indicating a burst effect. Between two and eight hours, the release rate slowed to approximately 57.0%, and it continued to maintain this level over 24 h, resulting in a total release of approximately 60.0%. This sustained release behaviour suggested that PLGA nanoparticles could retain therapeutic concentrations of PPIX over extended periods, which is a desirable feature for PDT. Encouraged by the previous results, da Silva and co-workers evaluated the cytotoxic activity of these nanoparticles in topical applications against murine melanoma B16-F10 cell cultures. Their findings underscored the dual benefits of PLGA nanoparticles: reducing dark cytotoxicity while maintaining the photodynamic efficacy of PPIX upon light activation [35]. The study revealed that PLGA encapsulation did not hinder the formation of singlet oxygen, the key mediator of PDT, upon light irradiation. Notably, PPIX-loaded nanoparticles demonstrated significantly lower cytotoxicity in the absence of light compared to the free drug. Approximately 49% of cells remained viable with free PPIX in the dark, while about 90.6% of cells survived when treated with the nanoparticle formulation, indicating that PLGA provided a protective effect against the cytotoxicity of PPIX in the dark. During PDT experiments, a higher light dose of 1500 mJ/cm^2^ showed that 3.91 μg/mL PPIX led to similar cell viability outcomes for both free and PLGA-encapsulated PPIX (approx. 34% viable cells), thus suggesting that encapsulation did not compromise PPIX photodynamic effect. At a higher concentration (7.91 μg/mL), phototoxicity increased for both formulations but was more pronounced with free PPIX, leaving only 5.8% of cells viable compared to 21.7% for the nanoparticle formulation [35]. Therefore, based on these findings, the protective nature of PLGA nanoparticles against dark cytotoxicity, while preserving PPIX’s photodynamic activity, can be highlighted.

Another treatment application where protoporphyrin IX encapsulated in PLGA nanoparticles can be employed is photodynamic antimicrobial chemotherapy (PACT), allowing for the eradication of bacterial and fungal species. The antimicrobial applications of PPIX-loaded PLGA nanoparticles are particularly important, as they broaden the therapeutic spectrum of this system beyond oncology. Recently, Izquierdo and colleagues have utilised PPIX/PLGA nanoparticles for photodynamic antimicrobial inactivation of *Staphylococcus aureus*, demonstrating the dual benefits of preserving PPIX’s photodynamic activity while enhancing its solubility and reducing cytotoxic effects on mammalian cells [36]. To fabricate these nanoparticles, the authors used an amine-terminated PLGA in a single-emulsion solvent evaporation method. TEM microscopy studies revealed that the PPIX-loaded nanoparticles were spherical, with a mean diameter of 33.6 nm, which was larger than that of the empty nanoparticles (16.9 nm). Zeta potential measurements at neutral pH showed similar electrokinetic potentials for both empty (−11.9 mV) and PPIX-loaded (−12.2 mV) nanoparticles. Encapsulation efficiency was calculated to be 13.7 wt.%, with a PPIX loading of 0.14 wt.%. Furthermore, in vitro microbiological tests demonstrated that both free and encapsulated PPIX resulted in a two-log reduction in *S. aureus* growth at the highest PPIX concentration tested. Interestingly, the PPIX-loaded nanoparticles demonstrated a slightly enhanced bactericidal effect, reducing bacterial growth by an additional 0.5 log compared to free PPIX. Furthermore, for potential topical applications, the cytotoxicity of PPIX/PLGA nanoparticles and free PPIX was assessed using fibroblast cell cultures, where the latter exhibited significant dose- and time-dependent cytotoxicity. In contrast, encapsulated PPIX showed no detectable cytotoxicity, underscoring the safety benefits of nanoparticle delivery systems [36].

#### 2.1.2. Protoporphyrin IX in Connection with Bioactive Substances

PLGA was not the only polymer used in formulations employing PPIX as a photosensitizer for PDT studies, but other bioactive substances were also applied, including hyaluronic acid, gelatin, chondroitin sulfate, and hydroxyapatite. These bioactive compounds, similar to PLGA, introduced additional biological functionalities, particularly for targeted therapy. The first one was employed by Wang and colleagues, who developed hyaluronic acid-block-poly(D,L-lactide-*co*-glycolide) (HA-b-PLGA) micelles encapsulating PPIX to create a drug delivery system of improved PDT specificity and effectiveness targeting CD44-overexpressing cancer cells [37]. The copolymer was synthesised using an end-to-end coupling strategy, where amino-functionalized hyaluronic acid was linked to the COOH terminal groups of PLGA. Structural confirmation of the copolymer was achieved through ^1^H NMR spectroscopy. Notably, when the copolymer was dissolved in D_2_O, the proton signals from PLGA disappeared, indicating the formation of core-shell micelles that shielded the PLGA chains. In due course, PPIX-loaded micelles (PPIX/HA-b-PLGA) were prepared using the solvent-dialysis method, with the critical micelle concentration (CMC) determined to be 4 mg/L via pyrene fluorescence assays. For comparison, PPIX-loaded PLGA nanoparticles (PPIX/PLGA-NPs) were synthesised using the nanoprecipitation method. TEM imaging revealed micelle diameters of approximately 150 nm, while DLS measured a particle size of 213.4 nm, a PDI of 0.152, and a zeta potential of −24.3 mV for the micelle suspension. The penetration and phototoxicity of PPIX/HA-b-PLGA micelles were assessed in A549 cancer spheroids. A549 spheroids were treated with targeted micelles, PPIX/PLGA-NPs, or free PPIX, followed by irradiation with 625–630 nm light at a dose of 3 J/cm^2^. PPIX HA-b-PLGA micelles demonstrated significantly enhanced phototoxicity at concentrations of 5 and 10 µmol/L, whereas cells treated with 1 µmol/L PPIX retained full viability. This effect was also observed in two-dimensional monolayer cell cultures and three-dimensional tumour spheroids. The authors concluded that the enhanced PDT effectiveness was attributed to improved cellular uptake and deeper penetration into the spheroid structures [37].

#### 2.1.3. Various Activation Approaches of Protoporphyrin IX in Connection with PLGA and Other Biopolymers

**Figure 4:**
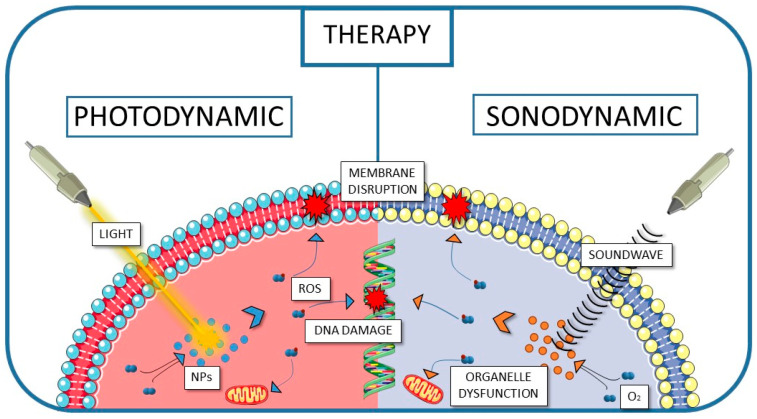
The schematic visualisation of the pathways of cancer cell destruction used in PDT and SDT.

PPIX in connection with PLGA and various biopolymers has also been subjected to diverse activation mechanisms, aiming to overcome the limitations of conventional light-triggered PDT. PPIX has to be activated, and the transfer of the absorbed energy is possible not only by light but also by ultrasound. Therefore, the utilisation of PLGA-based nanoparticles containing PPIX has also been assessed in sonodynamic therapy (SDT). Sonodynamic therapy uses ultrasound to activate specialised sonosensitizers, which then generate ROS within targeted cancer tissues. The mechanical and cavitation effects of ultrasound enhance the activation of sensitisers and promote localised ROS production [38,39]. These reactive oxygen species can destroy cancer cells by oxidising lipids and damaging the membrane, as well as disrupting DNA and affecting the function of organelles [40], similar to the ROS produced in the mechanism used in PDT (Figure 4).

To pursue this goal, He and co-workers explored a groundbreaking approach to treating osteochondral defects (OCDs) caused by giant cell tumours of bone (GCTB) by employing a double-layered scaffold made from shell-core nanofibers [41]. This innovative design was intended to allow a “spatiotemporal control,” enabling both targeted tumour treatment and precise osteochondral regeneration. The authors created shell-core nanofibers using coaxial electrospinning technology. The outer shell comprised protoporphyrin IX and gelatin (GT), while the inner cores included chondroitin sulfate (CDS)/PLGA or hydroxyapatite (HAp)/PLGA. These fibres were fragmented into nanoscale short fibres and combined with polyethene oxide and hyaluronan to produce inks for 3D printing. These materials have been applied for the preparation of a scaffold consisting of an upper PPIX/GT-CDS/PLGA layer and a lower PPIX/GT-HAp/PLGA layer. This type of structural complexity was crucial for simultaneously eradicating tumours and guiding tissue regeneration on a single platform. The scaffold’s properties were thoroughly characterised by various techniques. TEM and SEM analyses revealed that the GT-PLGA, PPIX/GT-CDS/PLGA, and PPIX/GT-HAp/PLGA nanofibers shared similar diameters (~0.4 μm), and the inks produced short nanofibers with diameters of 110–150 μm, averaging approximately 130 μm. The 3D-printed scaffold exhibited a cylindrical, porous structure (4 mm in diameter and height) with nanofiber diameters around 500 μm, tightly connecting the two layers. Next, this layered scaffold was implanted after tumour resection and subjected to ultrasound (2.5 MHz for 50 s), activating the PPIX for sonodynamic therapy. In the course of biological studies, SDT led to selective tumour damage. The prevention of recurrence was possible by producing a thermal effect that accelerated the release of bioactive factors—CDS and HAp—from the scaffold’s core. These factors promoted stem cell differentiation into cartilage and bone tissues, ensuring precise and timely regeneration at the OCD site. Biological assessments highlighted the scaffold’s efficacy. Ultrasound exposure significantly increased the temperature of PPIX-containing nanofibers compared to the control groups, demonstrating the thermal effects of PPIX during SDT. In vitro tests with GCTB cells confirmed that PPIX-containing scaffolds induced significant cytotoxicity after ultrasound treatment, resulting in a reduction in cell viability to approximately 10%. In contrast, control groups without PPIX showed negligible cytotoxicity, confirming the scaffold’s selectivity and safety [41]. In vivo evaluations on mice further validated the approach as tumours treated with PPIX-containing scaffolds were significantly smaller compared to those in non-PPIX groups, demonstrating the scaffold’s dual functionality in tumour suppression and tissue regeneration. The obtained results showed that the fabricated scaffold offered a synchronised approach to effective tumour eradication and precise osteochondral repair.

To overcome the PPIX limitation related to relatively low visible light activation wavelength (approx. 630 nm), which results in superficial tissue penetration, several attempts have been made to implement additional metallic nanoparticles to extend the activation to near-infrared (NIR) or even radio-frequency wavelengths. These hybrid systems were designed to exploit the deeper penetration of NIR and ionising radiation, thus broadening the therapeutic reach of PDT. The first region was achieved by Prieto and co-workers, who addressed the challenge of low tissue penetration in classic PDT by synthesising upconversion nanoparticles (UCNPs) that emit ultraviolet and visible light upon NIR excitation and co-encapsulating them with PPIX in PLGA-PEG nanoparticles. These UCNPs incorporated Nd^3+^ as a sensitiser to enable excitation at 808 nm [42]. UCNPs were obtained through solvothermal synthesis and exhibited a mean size of 41.1 nm as observed in TEM images. Elemental composition analysis by EDX confirmed the presence of NaYF_4_ (host matrix), Yb^3+^ (sensitiser), Tm^3+^ (activator and accumulator), and Nd^3+^ (energy transfer promoter for 808 nm absorption). PLGA-PEG nanoparticles encapsulating both UCNPs and PPIX were fabricated using a nanoprecipitation method with water and THF as miscible solvents, resulting in nanoparticles with an average size of 288.1 nm based on TEM images. Encapsulation efficiency was determined to be 6.24 wt.%, and drug loading was calculated at 0.22 wt.% using fluorescence spectroscopy and thermogravimetric analysis. The obtained UCNPs, upon NIR excitation, emitted both ultraviolet and visible light, efficiently activating the generation of ROS. PLGA-PEG nanoparticles containing both UCNPs and PPIX produced 3.4 times more ROS compared to those containing only PPIX. In the course of biological evaluation, photodynamic effects were assessed across multiple cell types, including melanoma cells (B16F1), mouse mesenchymal stem cells (mMSCs), human dermal fibroblasts, and macrophages. Fluorescence microscopy revealed oxidative damage in melanoma cells irradiated for 20 min at 808 nm, with a central area of cell death (visualised by ethidium bromide staining) surrounded by live cells (calcein staining). No significant cytotoxicity of PLGA-PEG nanoparticles (either with UCNPs alone or with both UCNPs and PPIX) on fibroblasts and keratinocytes was noted at tested concentrations (0.01–0.4 mg/mL). However, macrophage viability dropped below 70%, likely due to phagocytic activity as the authors presumed. Ultimately, ex vivo skin permeation studies using human skin and Franz diffusion cells demonstrated enhanced penetration of PPIX when encapsulated in PLGA-PEG nanoparticles. Unlike free PPIX, which remained on the outer skin layer, encapsulated PPIX permeated through the epidermis and reached the dermis after 24 h at room temperature [42].

**Figure 5:**
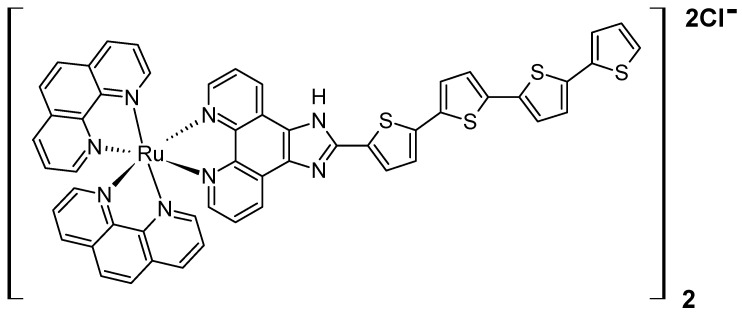
The chemical structure of ruthenium photosensitizer used by Azad and co-workers [45].

However, it was found that the deepest tissue penetration can be achieved when X-rays are used to activate adjacent photosensitizers, such as PPIX, via Förster resonance energy transfer (FRET), thereby generating reactive oxygen species to kill cancer cells. The so-called radioPDT employing protoporphyrin IX as the main PS has been investigated by several teams so far, all of them utilising LaF_3_:Ce^3+^ nanoscintillators (NSCs) for X-ray-triggered radioluminescence [43,44,45]. It is worth noting that compared to NIR-triggered systems, radioPDT takes a step further by harnessing deeply penetrating ionising radiation, potentially enabling treatment of other inaccessible tumours. In the first described study, using a wet-chemistry method in DMSO, Zhou and co-workers synthesised luminescent nanoscintillators composed of cerium-doped lanthanum(III) fluoride (LaF_3_:Ce^3+^), which were further incorporated into PLGA-based microspheres alongside protoporphyrin IX [43]. The preparation of the PLGA microspheres involved a modified emulsion/evaporation technique, followed by freeze-drying and lyophilisation for 24 h. The resulting microspheres were uniformly spherical with an average diameter of approximately 2 μm. When exposed to X-rays at 90 kV, the NSCs efficiently transferred energy to PPIX, generating singlet oxygen, which was subsequently tested on prostate cancer cells (PC3). The X-ray irradiation of the microspheres caused oxidative stress, mitochondrial damage, and DNA fragmentation in these cells. The biological evaluation also revealed that pure LaF_3_:Ce^3+^/DMSO/PLGA nanoparticles used as a control exhibited no significant toxicity to the PC3 cells. In contrast, the LaF_3_:Ce^3+^/PPIX microspheres reduced cell viability to approximately 60%, indicating a mild toxic effect likely due to the generation of singlet oxygen under X-ray activation. In similar studies, PEG-PLGA nanospheres were loaded with a scintillator (LaF_3_:Ce^3+^) and PPIX to enable radioPDT [44]. The scintillators were synthesised by reacting lanthanum(III) and cerium(III) nitrates with ammonium fluoride. Using a nanoprecipitation method, these NSCs, along with PPIX, were encapsulated within polymeric nanoparticles composed of PEG-PLGA. Characterisation using UV-Vis spectroscopy and electron microscopy confirmed a high encapsulation efficiency exceeding 90%. The nanoparticles exhibited stability for up to 24 h and demonstrated slow-release kinetics. In addition, TEM imaging revealed core-shell structures, with NSC sizes ranging from 10 to 50 nm, while PEG-PLGA nanoparticles measured 90–120 nm by DLS. The zeta potential ranged between −15 and −30 mV, ensuring colloidal stability. Further in vitro experiments using human skin fibroblasts (GM38) and prostate cancer cells (PC3) demonstrated significant cytotoxic effects. Under UV light irradiation (403 nm, 10 J/cm^2^), PC3 cell viability dropped to 15–20%, while X-ray irradiation (8 Gy) reduced viability to approximately 40%. In addition to cell studies, in vivo assessments were conducted using immune-competent C57BL/6 mice, where the radioPDT nanoparticles were administered intravenously via the tail vein. Histopathological analysis revealed no evidence of toxicity in major organs, including the lungs, liver, spleen, and kidneys. Confocal fluorescence microscopy confirmed PPIX uptake in various organs, with the spleen exhibiting the highest fluorescence intensity, followed by the liver and lungs. These results suggest that the nanoparticles selectively accumulated in specific tissues and could serve as a potential theranostic tool, combining therapeutic and diagnostic capabilities [44]. The same PPIX/NSCs/PEG-PLGA nanoparticles were synthesised and compared in terms of singlet oxygen generation and cytotoxicity on PC3 cell lines with a novel X-ray-triggered ruthenium photosensitizer encapsulated within pegylated PLGA nanoscintillators (Figure 5) [45]. Using a nanoprecipitation method, the authors encapsulated LaF_3_:Ce^3+^ NSCs and photosensitizers within PEG-PLGA, creating two formulations: Ru/radioPDT and PPIX/radioPDT nanoparticles.

The detailed physicochemical characterisation results revealed hydrodynamic diameters of 96 nm for PPIX-loaded particles and 118 nm for Ru-loaded particles. Zeta potential measurements showed distinct negative charges, with values of −17.4 ± 0.7 mV for Ru/radioPDT NPs, −27.4 mV for PPIX/radioPDT NPs, and −19 mV for bare NSCs. Stability tests over 24 and 48 h confirmed that the nanoparticles remained stable in physiological serum conditions without aggregation or degradation. To assess the effectiveness of PDT, the authors measured singlet oxygen production using the Singlet Oxygen Sensor Green (SOSG, ThermoFisher Scientific, Waltham, MA, USA). Under 405 nm light irradiation, Ru/radioPDT NPs generated more singlet oxygen than their PPIX counterparts. Despite this, both formulations induced significant PC3 cell death under light irradiation (402 nm, 2 J/cm^2^). When X-ray irradiation (3 Gy) was applied, cytotoxicity increased further for both formulations, with Ru/radioPDT achieving a 16% higher efficacy than PPIX/radioPDT. However, statistical analysis revealed that this difference was not significant, likely due to the relatively low radiation dose used.

#### 2.1.4. 5-Aminolevulinic Acid in Connection with PLGA and Other Polymers

**Figure 6:**
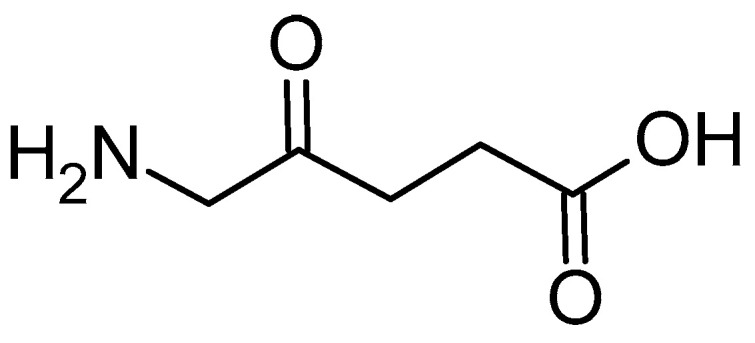
The chemical structure of 5-ALA.

5-Aminolevulinic acid (5-ALA, Figure 6) is a precursor for the in situ synthesis of protoporphyrin IX inside living cells. In brief, two molecules of 5-ALA undergo condensation by the enzyme ALA dehydratase (also known as porphobilinogen synthase) to form porphobilinogen (PBG). Four PBG molecules are then linked together by hydroxymethylbilane synthase to generate hydroxymethylbilane, which spontaneously cyclizes into uroporphyrinogen III under the action of uroporphyrinogen III synthase. This intermediate is sequentially modified by uroporphyrinogen decarboxylase and coproporphyrinogen oxidase, leading to the production of protoporphyrinogen IX. Finally, protoporphyrinogen IX is oxidised by protoporphyrinogen oxidase to yield protoporphyrin IX (PPIX) [31].

For PDT purposes, 5-ALA faces similar challenges as PPIX, including its poor stability, rapid elimination, weak bioavailability, and limited tumour cell penetration. Moreover, optimising its delivery is essential for maximising intracellular PPIX formation and, consequently, PDT efficacy. Therefore, PLGA nanoparticles appear to be a promising tool for alleviating these limitations. In this context, nanoencapsulation is intended not only to protect 5-ALA but also to modulate its spatiotemporal availability at the target site. The combination of 5-ALA and this polymeric delivery system was employed by Shi and co-workers, who developed 5-ALA-loaded PLGA nanoparticles using a modified double-emulsion solvent evaporation method [46]. The process began by dissolving PLGA in dichloromethane (DCM) to form the oil phase, while 5-ALA was dissolved in phosphate-buffered saline (PBS) at pH 5.0 to create the internal aqueous phase. These two phases were combined and subjected to sonication on ice using a probe sonicator in a discontinuous mode for 40 s at 120 watts, resulting in a primary emulsion. This emulsion was then transferred into an external aqueous phase consisting of 1% ALA in PBS (pH 5.0) and underwent a second round of sonication on ice for another 40 s. The resulting water-in-oil-in-water emulsion was stirred for four hours to evaporate the organic solvent, yielding a colloidal suspension of ALA-loaded PLGA nanoparticles. These were subsequently isolated by centrifugation and freeze-dried. The final nanoparticles were spherical, with a mean size of 65.6 nm and a polydispersity index (PDI) of 0.62. Encapsulation efficiency was 65.8%, whereas the 5-ALA loading capacity was 0.62%%. XRD analysis revealed that 5-ALA transitioned to an amorphous phase when encapsulated within the PLGA matrix. In the course of their biological activity evaluation, the nanoparticles demonstrated effective uptake by squamous cell carcinoma (SCC) cells, where they localised in the cytoplasm. Kinetic studies of PPIX fluorescence and MTT assays confirmed that the 5-ALA-loaded PLGA nanoparticles exhibited superior cytotoxicity compared to free 5-ALA at the same concentration. Using A431 SCC cell lines, the PDT parameters were set to 632.8 nm wavelength, a power density of 8.6 mW/cm^2^, and an energy density of 8 J/cm^2^. After 24 h of incubation with 5-ALA-loaded PLGA nanoparticles at a concentration of 2.7 mg/mL, cell viability decreased to approximately 20%.

The nanoparticles obtained using the same methodology were utilised by Wang and co-workers to treat male SKH-1 hairless mice that had been previously induced with cutaneous SCC cells using ultraviolet irradiation [47]. Moving from cell culture to a diseased-skin model allowed assessment of whether the formulation advantages persist under topical delivery conditions. To test a novel treatment, 5-ALA-loaded PLGA nanoparticles were lyophilised and mixed into an oil-in-water cream matrix to produce a formulation containing 0.8% of 5-ALA. This cream was applied topically to the skin of the tumour-bearing mice, and the fluorescence intensity of 5-ALA-induced PPIX was measured between 1 and 9 h post-application. Following the application of the cream, the fluorescence intensity of PPIX steadily increased from 1 to 6 h, reaching its peak at 6 h and then began to decline. Comparatively, when a neat 5-ALA cream of the same concentration was applied, PPIX fluorescence peaked earlier at 3 h and declined by 6 h. Despite the slower release of 5-ALA from the nanoparticle formulation, the relative PPIX fluorescence intensity in the 5-ALA PLGA NP group exceeded that of the neat 5-ALA group. During PDT treatment, the mice were irradiated with a helium-neon laser at 632.8 nm, with a power density of 8.6 mW/cm^2^ and an energy density of 15 J/cm^2^. In one experimental group, microneedling was performed prior to the application of the 5-ALA PLGA NP cream to enhance skin penetration. Multiple sessions of PDT using 5-ALA PLGA NP cream significantly inhibited tumour growth. Tumour sizes in the nanoparticle PDT group showed remarkable reductions, with significant shrinkage observed a week after the second treatment. After the fourth treatment, tumours were reduced by an average of 68% over a two-week period. In situ fluorescence examinations revealed that PPIX production was higher in both the ALA PLGA NP group and the microneedling combination group compared to the neat ALA cream group. However, statistical analysis indicated no significant difference between the nanoparticle and microneedling groups [47].

As previously described for PPIX-loaded HA-PLGA block copolymer, a similar approach was used to develop an innovative core-shell-structured dual-drug delivery system encapsulating 5-ALA [48]. The process began with the conjugation of hyaluronic acid (HA) and PLGA to produce an HA-PLGA block copolymer. Separately, 5-ALA was linked to PLGA via a pH-sensitive hydrazone bond derived from carboxyl phenylhydrazine (HBA), resulting in a PLGA-HBA-ALA functionalized copolymer. The final nanoparticles, designated HA-PLGA@ART/ALA NPs, were prepared using a self-assembly method in DMSO with artemisinin (ART) encapsulated in the PLGA core. This structure featured an HA and 5-ALA-containing shell and an ART-loaded core, designed to enable a sequential controlled release of 5-ALA and ART. Artemisinin is a natural compound derived from *Artemisia annua*, widely known for its potent antimalarial properties and emerging potential in cancer treatment. Its cytotoxic potential arises from an endoperoxide bridge, which generates ROS. In this system, 5-ALA was released first through the pH-sensitive cleavage of the hydrazone bond, generating PPIX and increasing heme production. The elevated heme levels enhanced the therapeutic effect of the subsequently released ART. This release mechanism was attributed to the breakdown of the hydrazone bond, which caused the nanoparticles to disintegrate, thereby facilitating the release of ART. In the physicochemical characterisation, TEM imaging revealed the particle size increase while NPs were loaded: HA-PLGA NPs measured 172.1 nm, HA-PLGA/ALA NPs 210.4 nm, and HA-PLGA@ART/ALA NPs 232.3 nm. Zeta potential measurements showed increasingly negative values of −20.8 mV, −25.6 mV, and −30.2 mV, respectively. These progressive size and surface charge shifts were consistent with the stepwise assembly of the shell and drug cargoes. Cellular uptake studies demonstrated efficient entry of HA-PLGA/ALA NPs into cells, with no significant difference in fluorescence intensity between free ALA and HA-PLGA/ALA NPs after four hours of incubation. To enhance the therapeutic efficacy of the obtained treatment system, sonodynamic therapy (2 W/cm^2^, 60 s) was applied. Cytotoxicity studies on human hepatoma HepG2 cell line showed similar cell viability reductions (approximately 20%) for ALA+ART and HA-PLGA@ART/ALA NPs when treated with ultrasound. However, in vivo experiments using HepG2 tumour-bearing mice revealed that HA-PLGA@ART/ALA NPs with ultrasound produced superior outcomes. After six administrations, the relative tumour volumes (V/V_0_) were as follows: control (5.12), ultrasound (US) alone (4.87), ART alone (3.75), ART+US (2.45), ART+ALA (2.08), ART+ALA+US (1.73), HA-PLGA@ART/ALA NPs (1.29), and HA-PLGA@ART/ALA NPs with ultrasound (0.77) [48].

The most sophisticated therapeutic system based on a comprehensive strategy integrating active and passive targeted delivery was proposed by Li and co-workers [49]. They combined the CC9 peptide, gold nanoparticles (AuNPs), and PLGA polymer into a single therapeutic approach. The process began with the synthesis of AuNPs via the trisodium citrate reduction method. Subsequently, 5-ALA and CSNIDARAC (CC9), a lung tumour-targeting peptide, were added to the mixture. Through Au-sulfur and ionic bonding, the ALA/CC9@AuNPs (ACNs) were formed. These nanoparticles were then encapsulated in PLGA carriers using an emulsion evaporation method, resulting in ACNPs. TEM imaging revealed uniformly dispersed AuNPs averaging 30.6 nm, with the ACNPs achieving a mean size of approximately 140 nm. Zeta potential measurements indicated increasing values from −29.9 mV for AuNPs to +10.11 mV for ACNPs, showing enhanced stability. DSC measurements confirmed that 5-ALA was chemically incorporated into the nanoparticles, and UV-Vis studies verified the interactions of AuNPs, 5-ALA, and CC9 within the PLGA matrix. Notably, ACNPs displayed pH-dependent controlled release of 5-ALA, with 69.5% released in a weakly acidic environment, mimicking tumour conditions.

Biological evaluations further demonstrated the activity of ACNPs. Haemolysis tests showed that ACNPs were safe for blood compatibility in PBS, with severe haemolysis only occurring in deionised water. Cellular transformation studies demonstrated the successful conversion of 5-ALA to PPIX, resulting in bright red fluorescence within 6 h of incubation. In vitro cytotoxicity tests revealed significant inhibition of lung cancer cell lines, particularly NCL-H460 cells, with viability reduced to 30% compared to 50% for A549 cells under 630 nm irradiation. ACNP-based PDT was found to kill H460 cells 1.4- and 2.3-fold more effectively than free 5-ALA at 50 and 100 μg/mL concentrations, respectively. Further in vivo studies highlighted the tumour-targeting capabilities of ACNPs. Biodistribution experiments in tumour-bearing mice showed gradual accumulation of ACNPs at tumour sites, with the strongest fluorescence signals observed 4 h after administration and persisting for over 6 h. Compared to 5-ALA, ACNPs exhibited greater tumour accumulation and reduced liver localisation, underscoring their enhanced targeting ability. Encouraged by these results, the authors tested the therapeutic efficacy of ACNPs on H460 tumour-bearing nude mice. The treatment hampered tumour growth by day 7 and initiated tumour shrinkage from day 7 to day 15. This anti-tumour effect, according to the authors, is driven by the enhanced permeability and retention (EPR) effect of ACNPs, which has been shown to be superior to that of 5-ALA alone [49].

#### 2.1.5. Concluding Remarks for PPIX and 5-ALA Encapsulation in PLGA

**Figure 7:**
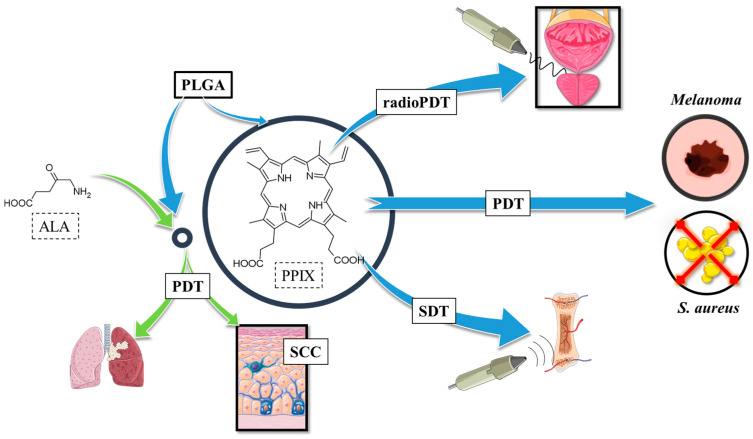
Applications of PPIX in polymeric nanoparticles; ALA—5-aminolevulinic acid, PDT—photodynamic therapy, PPIX—protoporphyrin IX, SDT—sonodynamic therapy, SCC—squamous cell carcinoma.

Considering the results of the research discussed above and schematically summarised in Figure 7, several conclusions and future remarks can be drawn regarding the improvement of PPIX and 5-ALA delivery and retention, reduced dark cytotoxicity, and maintained photodynamic activity. Additionally, enhanced targeting and uptake with functionalized nanocarriers, and expanding PPIX activation beyond visible light can be noted. Studies showed that PPIX/PLGA nanoparticles significantly improve PPIX penetration in the skin, increasing retention by 23 times in the stratum corneum and 10 times in the epidermis/dermis compared to free PPIX [33]. Furthermore, a controlled release behaviour was observed, with a burst release (~37% in the first 2 h), followed by sustained release (~60% over 24 h) [34]. In terms of PDT activity, PLGA encapsulation provides a protective effect against PPIX dark cytotoxicity. Whereas PLGA-encapsulated PPIX shows minimal toxicity before activation, at the same time, free PPIX exhibits high cytotoxicity even in darkness. In PLGA micelles, photodynamic efficacy is preserved, ensuring the production of singlet oxygen upon exposure to light. The polymer itself allows for functional modifications, such as the connection to hyaluronic acid, which improves PDT selectivity for CD44-overexpressing cancer cells [37]. These HA-PLGA micelles demonstrated enhanced cellular uptake and deeper tumour penetration, leading to higher phototoxicity in both 2D and 3D tumour models.

PLGA nanoparticles containing PPIX can be activated by both light and ultrasound. Studies showed selective tumour damage and synchronised osteochondral tissue regeneration when applied in a layered 3D-printed scaffold [41]. In addition, upconversion nanoparticles co-encapsulated with PPIX allow NIR excitation (808 nm), enabling deeper tissue penetration. These UCNP-based PLGA formulations improve ROS generation by 3.4x compared to PPIX alone [42]. Finally, LaF_3_:Ce^3+^ nanoscintillators in PLGA nanoparticles enable X-ray activation of PPIX, effectively treating deep-seated tumours through Förster Resonance Energy Transfer [43,44,45].

Future directions for PPIX/PLGA-based therapies rely on the combination therapies (e.g., PDT + SDT or PDT + immunotherapy), which may further improve treatment efficacy. Moreover, continued research into nanoparticle modifications (e.g., PEGylation, targeted ligands, co-delivery systems) can optimise specificity and reduce side effects. However, the still-not-addressed challenge remains scaling up for clinical use, requiring further studies on stability and large-scale synthesis. In conclusion, encapsulation of PPIX and 5-ALA in PLGA nanoparticles addresses key limitations of free compounds, enhancing solubility, selectivity, penetration, and safety, while maintaining or even improving their photodynamic and antimicrobial efficacy. Innovative activation methods (NIR, ultrasound, and X-ray) further expand its therapeutic potential in cancer and infectious disease treatment.

### 2.2. THPP in PLGA Nanoparticles

#### 2.2.1. THPP in Connection with PLGA

Another porphyrinoid photosensitizer commonly studied for incorporation into PLGA-based nanoparticles is 5,10,15,20-tetrakis(3/4-hydroxyphenyl)porphyrin (mTHPP/pTHPP). Building on the rationale established for PPIX, THPP encapsulation aims to mitigate aggregation, improve delivery, and enhance photocytotoxicity in a similar manner. The first examples of this approach were presented by Konan et al., who introduced a method for incorporating pTHPP into PLGA or PLA NPs [50]. Regarding the preparation procedure, targeted NPs were obtained using the emulsification-diffusion method. In brief, the appropriate polymer (50:50 PLGA, 75:25 PLGA, or PLA) and pTHPP were dissolved in benzyl alcohol, emulsified with an aqueous polyvinyl alcohol (PVA) solution, and stirred. Water was then added to allow diffusion of the benzyl alcohol. The suspensions were purified by filtration and freeze-dried in the presence of trehalose. The mean particle size and polydispersity index were assessed by photon correlation spectroscopy. The NPs made from copolymers exhibited similar properties: an average diameter of ~118 nm, a polydispersity index of 0.2, and drug loads of 7.8% (50:50 PLGA) and 8.2% (75:25 PLGA). The PLA NPs were larger (~125 nm) but had a smaller drug load (~7.3%) and a lower PDI (~0.16). The photocytotoxicity of NPs at different concentrations was tested on EMT-1 mammary tumour cells using an MTT assay. The activity of pTHPP on cells was observed 18 h after irradiation. A plateau was reached at a concentration of 12 mg/mL, where the lowest cell viability fraction (~10%) was observed. This may be explained by delayed cell death following damage. Among the formulations, the 50:50 PLGA NPs exhibited the highest phototoxicity on the EMT-1 cell line. The research suggests that the activity of pTHPP depended on the molar ratios and hydrophilicity of the polymers, and its toxic effect was strongly influenced by exposure time [50].

The follow-up study by Konan et al. focused on the properties of NPs themselves [51]. By decoupling materials variables from biological readouts, the authors clarified which formulation levers most strongly affect stability and handling. The goal was to achieve particles smaller than 200 nm. The study indicated that all the mentioned NPs exhibited similar characteristics in terms of particle size and zeta potential. The amount of loaded pTHPP did not depend on the type of polymer (PLGA copolymer or PLA). Additionally, higher theoretical drug loading increased the amount of pTHPP incorporated into NPs but decreased the entrapment efficiency. The mean size of NPs before freeze-drying was always slightly smaller than after drying in the presence of trehalose as a lioprotectant. In comparison, this process without trehalose resulted in significantly larger particles. For example, PLA NPs exhibited a significant increase in size, from approximately 100 nm before freeze-drying to around 700 nm after drying. It was also observed that the size of rehydrated freeze-dried NPs depends on the medium. The largest changes were observed when NPs were introduced directly into human plasma, whereas introducing NPs into distilled water resulted in only mild changes in diameter [51]. In the next study on the antitumor activity of pTHPP/PLGA or PLA nanoparticles, performed on EMT-6 mammary tumour cells, the NPs made from 50:50 PLGA/PLA copolymer revealed the highest cellular uptake, followed by 75:25 copolymer, PLA, and free pTHPP [52]. The uptake profile was consistent across all drug concentrations and was assessed using flow cytometry and fluorescence microscopy. Studies revealed that, with an increase in drug concentrations and incubation times, the number of captured particles also increased. Moreover, temperature influenced cellular uptake. At 4 °C, absorption was similar for all formulations and free pTHPP. However, at 37 °C, the uptake of the free drug increased slightly (1.5 times), whereas uptake for all three NPs increased threefold. To evaluate the impact of blood proteins on pTHPP photocytotoxicity, cells were irradiated at a light dose of 6 J/cm^2^. Then, cells were incubated in Waymouth growth medium supplemented with either 50% or 0% (*v/v*) fetal bovine serum (FBS). The MTT assay, performed 18 h after irradiation, revealed that incubation in medium without FBS resulted in high cell viability (80–100%) for 3 μg/mL and 6 μg/mL pTHPP. An exception was the 50:50 PLGA formulation, for which 70% cell viability was noted at 6 μg/mL. In the presence of 50% FBS, free pTHPP caused mild changes in viability at both concentrations. At lower concentrations, the 50:50 PLGA formulation slightly outperformed the others; however, all three NP formulations resulted in cell viability ranging from 40% to 60%. At higher concentrations, viability was similar across all three NP formulations. Fluorescence measurements of pTHPP indicated that particle efflux after irradiation was approximately 20%, compared to 60% for free pTHPP. Thus, PLGA-NPs with pTHPP encapsulated ensured drug retention within the cells [52].

A similar emulsification-diffusion method for the preparation of pTHPP/PLGA (50:50)-based nanoparticles was utilised by Vargas and co-workers [53]. Their photodynamic activity was evaluated using the chick embryo chorioallantoic membrane (CAM) model. Extending evaluation from monolayer cultures to a vascularized membrane enabled assessment of intravascular retention and responses. The NPs were purified through cross-flow filtration and freeze-dried in the presence of trehalose. The pTHPP loading, determined spectrophotometrically, was 7.8% *w/w*. The mean particle size was 117 nm with a PDI of 0.2. Minimal aggregation was observed when the NPs were stored with trehalose. Fluorescence angiography of the CAM model revealed that encapsulated pTHPP remained intravascular for a significantly longer period than free pTHPP. The free drug dissolved in ethanol extravasated from the vasculature faster than the NPs; their fluorescence was still present after 1500 s. The obtained NPs exhibited enhanced vascular occlusion in CAM compared to free pTHPP under equivalent light doses of 10, 15, and 20 J/cm^2^. The NPs induced more significant damage to the embryos, resulting in better photodynamic effects at lower drug concentrations and lower light doses compared to free pTHPP. The pTHPP NPs appear to have superior properties compared to solubilised pTHPP, likely due to their reduced diffusion out of the CAM vessels [53]. Several years later, in another study by Vargas et al., tetrahydroxyphenyl porphyrin derivative, mTHPP, was encapsulated into PLGA NPs via the previously described emulsification-diffusion method [54]. During the preparation process, an organic solution containing mTHPP, PLGA, benzyl alcohol, and propylene carbonate was added under mechanical stirring to an aqueous phase containing PVA (6%, 9%, or 17% *w/w*) as a stabilising agent. The addition of water triggered the formation of nanoparticles. The NPs were purified and freeze-dried with trehalose. Ultimately, NPs with diameters of 100 nm, 300 nm, and 600 nm were obtained. Larger particles contained lower amounts of residual PVA. Drug loadings across particle sizes were 9.5% for 600 nm NPs, 8.7% for 300 nm NPs, and 7.8% for 100 nm NPs, whereas the corresponding polydispersity indexes were 0.08, 0.05, and 0.04, respectively. X-ray diffraction confirmed that encapsulated mTHPP was amorphous. Larger NPs exhibited a significant reduction in ROS formation, even after prolonged exposure to light. The 100 nm NPs demonstrated superior photosensitizer effects, even at concentrations two times lower than those used for larger particles. This suggests that ROS production was dependent on mTHPP concentration or could be attributed to the higher surface area of the smaller NPs. In vivo analysis was also conducted using the CAM model. Smaller NPs (100 nm) achieved superior vascular occlusion compared to larger NPs or free mTHPP, with light doses of 15 J/cm^2^. It was also observed that residual PVA content was associated with increased mTHPP release from the NPs, and NP size correlated with differences in photosensitizer release. This study highlights that PVA plays a role in modulating the release of mTHPP from the NPs [54].

#### 2.2.2. THPP in Connection with Various Polymers

Grünebaum et al. employed the same compound, mTHPP, for photodynamic therapy of cholangiocarcinoma [55]. Tests on various compositions of polymer carriers brought significant information on their usability in related formulation studies. Three NP formulations were prepared using different polymers: 50:50 PLGA, PLA, and poly(butyl methacrylate-co-(2-dimethylaminoethyl) methacrylate-*co*-methyl methacrylate) 1:2:1 (Eudragit E^®^). PLGA and PLA were dissolved in organic solvents with mTHPP, mixed with a polyvinyl alcohol (PVA) solution, and stirred to evaporate the organic solvent. In contrast, Eudragit E^®^ was mixed only with an aqueous PVA solution. The suspensions were centrifuged and redispersed in distilled water. Photon correlation spectroscopy was used to determine the average diameter, PDI, and zeta potential of the NPs, while drug loading was quantified using an HPLC-DAD system. mTHPP- Eudragit E^®^-NPs were the smallest (~214 nm) with a positive zeta potential (+55 mV), whereas mTHPP-PLA-NPs were larger (~286 nm) and negatively charged (−37.1 mV). Drug loading was the highest in Eudragit E^®^ particles (163.3 µg mTHPP/mg NP) and the lowest in PLA NPs (93.1 µg mTHPP/mg NP). mTHPP-PLGA-NPs exhibited intermediate characteristics with a diameter of ~246 nm, a drug load of ~102.8 µg mTHPP/mg NP, and a zeta potential of −41.8 mV. The PDI ranged from 0.06 for mTHPP-Eudragit E^®^-NPs to 0.16 for PLA-based NPs (0.07 for mTHPP-PLGA-NPs). Release studies demonstrated minimal loss of mTHPP from Eudragit E^®^-based particles. Drug uptake studies conducted on bile duct carcinoma (TFK-1 and EGI-1) cell lines showed that mTHPP- Eudragit E^®^-NPs were superior to other formulations. Phototoxicity tests revealed that EGI-1 cells were more sensitive, with EC_50_ values of 0.3 µM and 0.6 µM for TFK-1 for Eudragit E^®^ and 0.2 µM (EGI-1) and 0.52 µM (TFK-1) for PLGA-based NPs following light irradiation (5 J/cm^2^). Caspase-3 activation tests demonstrated that PLGA NPs presented a superior photodynamic effect at concentrations ≤ 0.75 µM mTHPP. On the other hand, for PLA NPs, a significant increase in activity was observed only at a concentration of 5 µM mTHPP. LDH release results further confirmed the superiority of PLGA over the other NPs and, thus, PLGA appears to be the most promising choice for the treatment of cholangiocarcinoma [55]. Taken together, these findings indicate that cationic carriers may favour uptake, whereas PLGA balances loading, release, and cytotoxic mechanisms across relevant dose ranges.

Despite utilising bare PLGA polymeric materials with diverse lactic and glycolic acid monomers (75:25 or 50:50), researchers have also explored functionalized polymers, achieving enhanced physicochemical properties, better stability, and greater loading of fabricated PLGA-based nanoparticles. Pursuing these goals, Pramual et al. developed and characterised core-shell nanoparticles where PEGylated PLGA or PHBV (poly(3-hydroxybutyrate-co-3-hydroxyvalerate)) served as the inner polymer core, while lecithin and DSPE-PEG-COOH (1,2-distearoyl-*sn*-glycero-3-phosphoethanolamine-N-[carboxy(polyethylene glycol)-2000]) formed the outer lipid-PEG shell [56]. pTHPP was dissolved in acetonitrile with both polymers, whereas lecithin and DSPE-PEG-COOH (7.5:2.5, molar ratio) were dissolved in an aqueous ethanol at 65 °C. The lipid-aqueous solution was added to the polymer solution in a dropwise manner during stirring, allowing the NPs to self-assemble. The resulting solutions were washed with water and filtered. The particles were characterised by DLS for size and electrophoretic mobility for zeta potential. Depending on the initial drug loading, values were slightly different. The size of PLGA NPs ranged from 88 to 95 nm, with a zeta potential of −43 to −49 mV. PHBV NPs were much larger (213–230 nm), with a zeta potential of −35 to −40 mV. PHBV NPs exhibited slightly better drug loading, but PLGA NPs showed superior entrapment efficiency (~10% higher). PHBV NPs had higher polydispersity indices (0.10–0.14) compared to PLGA NPs (0.06–0.11). TEM images confirmed that the NPs were individual spherical particles. In addition, XRPD (X-Ray powder diffraction) revealed the amorphous form of pTHPP in the NPs. Cellular uptake studies on FTC-133 human thyroid carcinoma cells showed significantly higher uptake for PLGA NPs compared to PHBV and free pTHPP. Moreover, in the photocytotoxicity studies, significantly worse effects were observed for free pTHPP than for NPs. PLGA NPs demonstrated rapid action, making them suitable for conditions requiring immediate effects, while PHBV NPs may be considered better suited for cases requiring prolonged treatment [56]. These comparisons suggested that finer particle size and tighter distributions of PLGA cores translate into faster cellular engagement and earlier ROS-driven damage.

The same research group fabricated PLGA-lipid hybrid NPs loaded with pTHPP (pTHPP-PLHNPs) and examined them for overcoming drug resistance in lung cancer [57]. The NP size, determined by nanoparticle tracking analysis, was ~70.4 nm, with an entrapment efficiency of 88.91% and zeta potential of −39.2 mV. The in vitro studies were performed on A549 human lung adenocarcinoma and A549RT-*eto* multidrug-resistant (MDR) human lung adenocarcinoma cell lines using MTT assays and fluorescence microscopy. Cellular uptake assays demonstrated that pTHPP was poorly absorbed by cells. On the contrary, both cell lines absorbed approximately 0.8 mmol pTHPP/106 cells when the photosensitizer was encapsulated in PLHNPs. Moreover, a higher generation of superoxide anions was observed in cells treated with NPs after light irradiation (3 h; 6 J/cm^2^) compared to the free drug. Both cell lines showed similarly low resistance to the phototoxic effect, with IC_50_ values of 0.25 µM for A549 cells and 0.21 µM for MDR cells after light irradiation and MTT assay following 72 h. A significant increase in the percentage of apoptotic cells was observed at concentrations of 0.3 µM pTHPP-PLHNPs and higher. Free pTHPP demonstrated minimal toxicity toward cells. Moreover, pTHPP-PLHNPs showed similar effectiveness in killing both floating and substrate-attached A549 cells, with IC_50_ values of 0.13 µM and 0.14 µM, respectively [57].

In 2021, Forouharshad and Ajalloueian proposed a modified nanoprecipitation method to obtain stereocomplexed-PLA and pTHPP-loaded PLA NPs encapsulated into PLGA nanofibers for sustained and delayed release, addressing the limitations of poor encapsulation efficacy [58]. This complex architecture (drug in NPs and NPs in fibres) was intended to decelerate burst release while preserving the dose over days. The nanoprecipitation technique was used to prepare PLA and PLA NPs. The size was determined by polymer concentration, with increased polymer concentration resulting in increased size, and an average diameter ranging from 139 to 317 nm. The nanoparticles were characterised with FTIR and DSC analyses, confirming the creation of homo-crystals and stereocomplex crystals. The entrapment efficiency and drug loading content decreased with increased concentration of polymer, but the stereo-crystals had no negative effect on EE and DLC%. The drug-loaded PLA NPs encapsulated into PLGA nanofibers were obtained using the electrospinning method. The DLS registered for PLGA fibres was 546 nm. The addition of porphyrin did not change the morphology and size of the fibres, whereas adding sc-NPs increased the average diameter to 635 nm, and the addition of NPs-porphyrin increased the diameter to 896 nm. The drug release study confirmed that stereocomplex crystallinity is more resistant to degradation; however, due to the porous structure, 97% of the porphyrin was released within the first 24 h. In the case of PLGA/NPs-porphyrin, only 20% of the drug was released after 24h, indicating time-sustained release for 7 days. Concluding that loading drugs into nanoparticles and further encapsulating them is a promising technique for time-sustained release, which can occur over weeks rather than hours [58].

One of the major expectations associated with nanoparticles in terms of bioavailability is their ability to pass through the mucus present in the digestive system. Accordingly, surface functionalization (PEGylation vs. chitosan coating) was explored to tune muco-penetration and muco-adhesion. The study by Mahlert et al. focused on the development of PLGA-based NPs functionalized with PEG or chitosan (CS), containing mTHPP as a photosensitizer [59]. Particles were obtained by dissolving PLGA in acetone and adding mTHPP. In the next step, the solution was introduced into an aqueous stabiliser solution of PVA. The suspension was stirred, followed by the evaporation of acetone. The nanoparticle suspension was purified by centrifugation and then redissolved in water to obtain the final product, mTHPP-PLGA-NPs. For comparison purposes, chitosan-modified NPs were prepared using the same process. In this case, a stabiliser solution containing PVA and chitosan hydrochloride was used, resulting in mTHPP-CS-PLGA-NPs. By replacing neat PLGA with PLGA-PEG, PEGylated NPs (mTHPP-PLGA-PEG-NPs) were produced. Characterisation involved particle size and distribution measurements by DLS, laser Doppler electrophoresis for zeta potential, and HPLC for drug quantification. The functionalized NPs demonstrated distinct physicochemical characteristics: PEGylated NPs were smaller (~93 nm) with a less negative zeta potential (−19.8 mV), whereas CS-coated particles were larger (~120 nm) and positively charged (+10.3 mV). Drug loading was higher in PEGylated particles (66.5 μg mTHPP/mg NP) and lower in chitosan-coated NPs (43.8 μg mTHPP/mg NP). Unmodified particles were characterised by intermediate values: a diameter of ~108 nm, a zeta potential of −29.7 mV, and an intermediate drug load of ~51.8 μg mTHPP/mg NP. The polydispersity index ranged from 0.04 for PEGylated NPs to 0.20 for CS-based ones (0.07 for unmodified NPs). Biological studies were conducted on HT-29 monocultures and mucus-producing HT-29-MTX cultures. Phototoxicity studies showed slightly higher EC_50_ values for HT-29-MTX cells, indicating hindered transport of NPs through the mucus. The uptake of photosensitizers by HT-29 and HT-29-MTX cells was also evaluated, demonstrating that the presence of a mucus layer surrounding the cells impeded absorption. Additionally, higher absorption of mTHPP from PEGylated NPs was observed in mucus-covered cells compared to uncovered ones. Similar research conducted by Anderski et al. demonstrated comparable effects [60]. In this case, the Caco-2 cell line with biosimilar mucus was used. The mucus was a product of mixing PAA-mucin-gel, a lipid mixture (containing cholesterol, oleic acid, phosphatidylcholine, polysorbate 80, HEPES buffer), and bovine serum albumin (BSA). Once again, the mTHPP-PLGA-PEG-NPs exhibited the highest penetration through the mucus layer, reaching a maximal depth of 1600 μm. In comparison, the mTHPP-CS-PLGA-NPs penetrated mucus slightly (max. 200 μm). Cellular uptake of the mTHPP-PLGA-PEG-NPs into Caco-2 cells was the highest in the absence (approximately 5.6 μM mTHPP/cell) and presence (approximately 2 μM mTHPP/cell) of biosimilar mucus. Interestingly, free mTHPP showed similar uptake values (~5 μM and ~2 μM mTHPP/cell). Taken together, research by Mahlert et al. and Anderski et al. demonstrated that PEGylation of PLGA-based NPs enables better mucus penetration and higher photosensitizer uptake compared to non-PEGylated NPs. Therefore, PEGylation can be considered an effective strategy for enhancing drug delivery in photodynamic therapy for gastrointestinal cancers. This is likely due to the smaller diameter and higher hydrophilicity of PEGylated NPs. On the other hand, chitosan-modified NPs were characterised by the highest adsorption to mucus, likely due to their positive zeta potential, whereas mucus is negatively charged. Although free mTHPP demonstrates similar behaviour to PEGylated NPs, it is unsuitable for oral administration due to its low solubility in aqueous media [59,60].

mTHPP has also been studied as an imaging agent for monitored phototherapy. The traceable complex mTHPP-Pd was obtained after mTHPP metalation with Pd(II) and further characterised by laser ablation-inductively coupled plasma-mass spectrometry (LA-ICP-MS) [61]. The emulsion diffusion method was further used to embed mTHPP-Pd into nanoparticles. A PLGA solution was mixed with mTHPP-Pd in EtOAc, emulsified with PVA and stirred to evaporate EtOAc. The average diameter of the NPs was approximately 250 nm (PDI < 0.1), as determined by DLS. The assessment of tumour cells imaging was performed on the TFK-1 cell line (15-day-old tumour spheroids). Cells were incubated with mTHPP-Pd PLGA NPs or with mTHPP-Pd alone for 24 and 48 h. The distribution of mTHPP-Pd was analysed using LA-ICP-MS. NPs provided an even distribution of the complex in the outer parts of the spheroid. On the contrary, bare mTHPP-Pd accumulated unevenly, likely due to the hydrophobic nature of the drug or its partial precipitation. The authors were unable to detect the photosensitizer entrapped in PLGA NPs within the tested concentration range using fluorescence microscopy [61]. This highlights a practical advantage of ICP-based mapping for non-fluorescent or quenched formulations, where optical readouts may underestimate payload localisation.

#### 2.2.3. Concluding Remarks on the THPP Connection with PLGA

**Figure 8:**
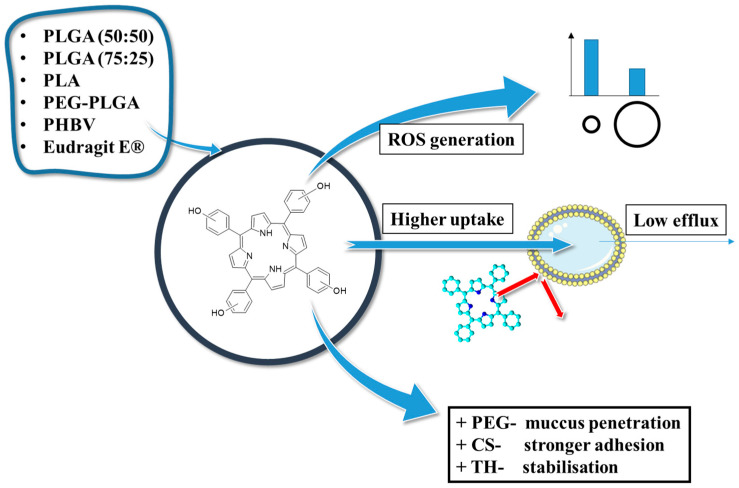
THPP in polymeric nanoparticles—properties and applications; PEG—polyethylene glycol, CS—chitosan, TH—trehalose, ROS—reactive oxygen species, mTHPP/pTHPP—5,10,15,20-tetrakis(3/4-hydroxyphenyl)porphyrin.

In conclusion, the studies on the characteristics, bioavailability, and photodynamic activity of THPP/PLGA NPs highlighted three key findings (Figure 8). First, in all cases, PLGA-based nanoparticles provided efficient encapsulation and sustained release of the photosensitizer, leading to enhanced photodynamic effects. Second, the physicochemical properties of nanoparticles, including size, polydispersity, and surface charge, significantly influence cellular uptake, phototoxicity, and drug retention. Third, functionalized polymers, such as PEGylated PLGA, improved nanoparticle stability, bioavailability, and penetration through biological barriers, demonstrating their potential for enhanced drug delivery in PDT and PDD.

### 2.3. Chlorin e6 in PLGA Nanoparticles

#### 2.3.1. Chlorin e6 in Connection with PLGA

Chlorin e6 (Ce6) is a highly efficient photosensitizer widely used in photodynamic therapy due to its strong absorption in the red-light spectrum (~660 nm), high singlet oxygen yield, and preferential accumulation in tumour tissues [62,63]. Its amphiphilic nature enhances cellular uptake and bioavailability, making it a promising candidate for cancer treatment. Up to date, Ce6 has been incorporated into various nanocarriers, such as liposomes, micelles, and polymeric nanoparticles, to enhance its stability, solubility, and targeted delivery [64]. Additionally, Ce6-based PDT has shown potential in antimicrobial applications, effectively inactivating bacteria and viruses with minimal side effects on healthy tissues [65].

Given these attributes, pairing Ce6 with PLGA seeks to stabilise the payload while tuning circulation, tumour deposition, and light-triggered response. In the study by Lee et al., Ce6-PLGA-based NPs were synthesised for luminescence resonance imaging and photodynamic therapy [66]. Firstly, the photosensitizer was conjugated with the polymer using the Steglich esterification method. Ce6 and DCC (*N*,*N*′-dicyclohexylcarbodiimide—a carboxylic group activator) were added to a solution of PLGA in DCM containing DMAP (4-dimethylaminopyridine), TEA (triethylamine), and pyridine, followed by stirring, filtration, and lyophilisation. The product was then poured into DMSO and dialysed to remove unbound Ce6. A similar process was used to obtain methoxyPEGylated PLGA (PLGA-mPEG). Secondly, a multiple-emulsion w_1_/o/w_2_ (water-in-oil-in-water) method was employed to obtain the NPs. Additionally, another batch of PLGA-Ce6 NPs was combined with an aqueous suspension of Fe_2_O_3_ for use in magnetic resonance imaging (MRI). The particles were subsequently washed with PBS and freeze-dried. This formulation was encapsulated in methoxyPEGylated PLGA NPs obtained via a similar process as the previous Ce6-PLGA-mPEG NPs. Regarding the latter, three formulations were prepared with different PLGA-mPEG:PLGA-Ce6 ratios (1:4, 1:1, and 0:1, respectively). The average particle diameter of the mPEGylated NPs was approximately 160 nm. However, NPs synthesised without PLGA-mPEG exhibited a tendency to aggregate and form larger particles (~2 μm). Non-mPEGylated particles were also significantly less stable. Zeta potentials of all combinations ranged from −4.1 mV to −3.3 mV, indicating that the presence of mPEG had minimal influence on the surface charge. Phototoxicity tests (light intensity 5.2 mW/cm^2^) were performed on human nasopharyngeal epidermal carcinoma KB cells. PLGA-mPEG NPs exhibited significantly greater toxicity toward cells compared to particles without PEG groups, achieving approximately 10% lower cell viability than free Ce6. In vivo assays conducted on KB tumour-bearing nude mice revealed that PLGA-mPEG NPs exhibited a strong fluorescent signal at the tumour site, as well as in the liver and kidneys. Free Ce6 accumulated in the kidneys and displayed much weaker fluorescence at the tumour site. Additionally, MRI demonstrated that NPs successfully darkened the tumour site, differentiating it from the background. Tumour regression studies showed that even at lower concentrations (0.1 mg/kg body weight), the NPs were more effective at inducing tumour size reduction than free Ce6 at higher concentrations (2.5 mg/kg body weight) [66].

#### 2.3.2. Chlorin e6 in Connection with PLGA in PLGA-Based Modalities

Beyond simple drug carriage, Ce6–PLGA platforms have been adapted into multifunctional modalities that combine imaging, catalysis, or modulation of the microenvironment to enhance PDT. Regarding the magnetic resonance purposes, the fabrication of iron-based NPs for MR imaging-guided ferroptosis with chlorin e6 to enhance photodynamic treatment of cancer was performed by Chen and co-workers [67]. Again, Fe_3_O_4_ was used as a contrast agent for imaging. In the preparation procedure, FeCl_3_·6H_2_O and iron powder were added to a hexane solution containing oleic acid (OA) as a surfactant, and the mixture was sonicated to ensure thorough mixing. Then, laurylamine was added, resulting in a brown solution, followed by heating, precipitation with ethanol, and separation by a magnet. The particles were redispersed in hexane, and (OA)-Fe_3_O_4_ NPs were obtained. Then, these NPs were dispersed in an organic phase, and citric acid was added. The mixture was stirred, precipitated, and washed with acetone. The OA-Fe_3_O_4_ NPs and Ce6 were introduced into a PLGA-DMSO solution. The mixture was stirred and dialysed, yielding Fe_3_O_4_-PLGA-Ce6 NPs. The targeted NPs exhibited a spherical morphology with an average diameter of 85 nm, as observed by TEM (approximately 100 nm in diameter, as measured by DLS), and a zeta potential of −30.1 mV in PBS. The loading contents were 25.5% for Fe_3_O_4_ and 22.1% for Ce6. The nanoparticles showed strong magnetic properties and were effective as T2-weighted MRI contrast agents. Drug release studies demonstrated pH-dependent release, with faster release occurring under acidic conditions, which mimics the tumour microenvironment. In vitro tests on 4T1 cells showed that Fe_3_O_4_-PLGA-Ce6 NPs had enhanced cytotoxicity. Cell viability was assessed using a CCK-8 assay, which showed the superiority of Fe_3_O_4_-PLGA-Ce6 NPs over free Ce6. After 24 h of laser irradiation, cells treated with Ce6 showed a reduction in cellular viability to 40%, while NPs lowered this to 25%. In vivo tests on 4T1 tumour-bearing mice demonstrated enhanced tumour targeting and retention in comparison to the free drug. The NPs showed synergistic photodynamic therapy and ferroptosis effects. As the authors concluded, the obtained Fe_3_O_4_-PLGA-Ce6 NPs were characterised by high biocompatibility, avoiding significant harm to healthy tissues [67].

Following a diagnostic thread, Huang et al. tested phototherapy guided by bimodal imaging for the treatment of uveal melanoma [68]. Compared with the previous Fe_3_O_4_–PLGA–Ce6 system, an Fe^3+^–tannic acid shell adds photoacoustic contrast and photothermal capability, enabling image-guided combination therapy. The multifunctional NPs were synthesised by loading chlorin e6 into PLGA NPs and wrapping Fe^3+^-tannic acid (Fe^3+^-TA) on the outer shell, resulting in FTCPNPs. First, PLGA and Ce6 organic solutions were mixed and sonicated to form an emulsion, to which a PVA solution was added, followed by the addition of isopropanol. The resulting mixture was then stirred, centrifuged, and washed with water, leading to PLGA/Ce6 NPs. These particles were subsequently added to a tannic acid solution, followed by the addition of FeCl_3_ and aqueous NaOH to adjust the pH to 7, forming FTCPNPs. The size of the NPs, measured by TEM, was 233 nm before and 246 nm after coating with Fe^3+^-TA. The PDI was low, at 0.086 for FTCPNPs and 0.036 for CPNPs, indicating a monodisperse character of the suspensions. The zeta potential of CPNPs was −18.57 mV, and was reduced to −29.70 mV after coating. The encapsulation efficiency of Ce6 was 82.1%, and the drug loading was approximately 4.6%. In vitro tests conducted on C918 human choroid melanoma cells and the ARPE-19 adult retinal pigment epithelial cell line using the traditional CCK-8 protocol revealed that, individually, photothermal and photodynamic therapies reduced cell viability to 40–50%. However, when combined, these methods reduced cell viability to approximately 10%. An in vivo assay conducted on tumour-bearing mice showed that the synergistic effect of photothermal and photodynamic therapies resulted in a complete reduction in tumour volume after two days. Phototherapies individually also demonstrated the ability to suppress tumour growth, but not as effectively as in a dualistic approach. Finally, magnetic resonance/photoacoustic (MR/PA) bimodal imaging demonstrated a strong signal intensity from the spleen and liver, with the strongest signal detected in the tumour, highlighting its potential for effective tumour localisation [68]. This underscores the value of integrating therapy with real-time readouts to time illumination and track response.

An interesting approach to photodynamic therapy of tumour cells based on metal–organic framework (MOF), Ce6, and PLGA was applied by Liu et al., where the NPs’ shell was synthesised from polymer and functionalized MOF [69]. Here, a TA/Fe MOF and glucose oxidase (GOx) were leveraged to reshape the tumour microenvironment (depleting glucose and tuning redox conditions) to potentiate Ce6 activity. The MOF consisted of tannic acid (TA) and iron ions and was obtained by mixing Ce6, FeCl_3_, and PLGA, followed by the addition of this solution to an aqueous mixture of TA and GOx under constant sonication, thereby creating the PTFCG solution. Next, PTFCG was mixed with poly(allylamine hydrochloride) and hyaluronic acid (HA) to create PTFCG-M NPs and PTFCG-MH NPs, respectively. The diameter and zeta-potential of NPs were measured using a Zetasizer. PTFCG NPs were the smallest in size (~175 nm) with the lowest zeta potential (−33.4 mV), while the PTFCG-M NPs had a positive potential (+17.3 mV). PTFCG-MH NPs were the largest (~205 nm) and had a negative zeta potential (−21.7 mV). The drug loading for Ce6 and GOx was 108.4 μg and 24.6 μg per mg of PTFCG-MH, respectively. The release assay demonstrated a glutathione (GSH) responsive release of the photosensitizer from NPs (20% release without GSH and 60% in the presence of GSH). The singlet oxygen generation studies revealed that PTFCG-MH had better performance in ROS generation than PTFCG NPs without the MOF. Cellular uptake was tested on MDA-MB-231 cancer cells. Nile red (NR) dye was added to NPs to facilitate tracking the absorption process. PTFCG NPs were superior in this test to PTFCG-MH NPs. Moreover, the concentration of intracellular ATP was measured using an ATP assay kit and Ellman’s reagent. It revealed that free GOx could decrease ATP levels by blocking the energy supply. Likewise, PTFCG-MH could also inhibit ATP generation. All formulations showed a decrease in cell viability when combined with laser irradiation. During in vivo tests, MDA-MB-231 tumour-bearing mice were treated with PTFCG-MH NPs and free Ce6. NPs showed higher specificity in binding to tumours than free photosensitizers and much stronger suppression of tumour growth [69].

Liang et al. investigated a multimodal PDT-chemotherapeutic approach by combining two cooperating agents in PLGA NPs: chlorin e6 and doxycycline (DOX) [70]. The rationale was to pair light-activated ROS with a cytotoxic payload to broaden mechanisms of action within a single carrier. The NPs were obtained by the double emulsion-solvent evaporation method. An aqueous DOX solution was mixed with an organic PLGA solution, sonicated, and followed by the addition of bovine serum albumin (BSA) to coat the NPs’ surface. The resultant double emulsion was dispersed dropwise into water, creating DOX-loaded NPs, denoted as DOX/PB NPs (doxycycline/PLGA-BSA nanoparticles). Then, a Ce6 organic solution was added to allow the absorption of the photosensitizer onto the surface of the NPs. The mixture was centrifuged, washed with water, and then freeze-dried, resulting in DOX/PB-Ce6 NPs as the final product. The hydrodynamic diameter of the NPs was measured using microscopic methods. In DMEM containing 10% FBS, the DOX/PB NPs ranged from 105.71 nm to 458.67 nm, whereas the DOX/PB-Ce6 NPs were larger due to the Ce6 deposition on the core-shell structures (164.18 nm–531.17 nm). The NPs revealed negative zeta potential values of −28.7 mV and −38.4 mV (for DOX/PB and DOX/PB-Ce6 NPs, respectively). Drug loading of DOX in both formulations was about 1.9% with approximately 55% entrapment efficiency, whereas the same parameters for Ce6 in NPs were 1.38% and 46%, respectively. The release assay revealed that drug release depended on pH; the lower the pH, the higher the percentage of drug released. For Ce6 in DOX/PB-Ce6 NPs, 32% remained in NPs at pH 5, whereas 47% at pH 7.2. A similar tendency was observed for DOX, with 21% left at lower pH and 37% at higher pH after 24 h in PBS. In vitro tests were conducted on HeLa and MCF-7 human breast cancer cells. These tests revealed the superiority of NPs with Ce6 after light irradiation in reducing cell viability and demonstrated enhanced apoptotic properties compared to DOX/PB. To assess hemocompatibility and the feasibility of intravenous administration, a haemolysis assay was conducted. However, it revealed that the NPs with Ce6 could destroy more red blood cells than DOX/PB. Nonetheless, the haemolytic percentages of the DOX/PB-Ce6 NPs were less than 3.5%, even at a concentration of 1.0 mg/mL, which indicated good blood compatibility. In conclusion, this research has shown that chemo-photodynamic synergetic formulations, such as the one investigated, may demonstrate higher therapeutic effectiveness than chemotherapy alone [70].

**Figure 9:**
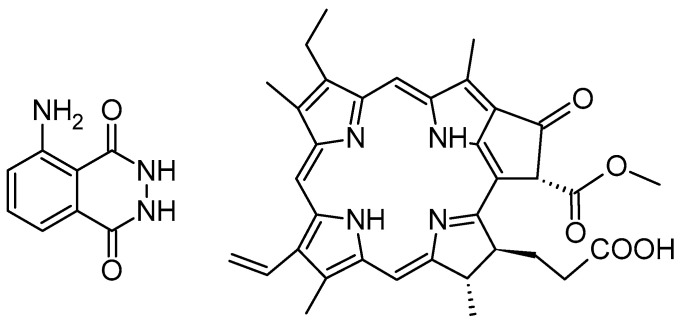
The chemical structures of luminol (**left**) and pheophorbide *a* (**right**).

Despite utilising doxycycline in combination with Ce6 for PDT-chemotherapeutic treatment of cancer, another compound was encapsulated with chlorin e6 by Lv et al. [71]. They investigated PLGA-based NPs containing the Ce6 photosensitizer and luminol (Lum, Figure 9) for the control of inflammation and infection in mice. Luminol is a dye which undergoes a chemiluminescent reaction when it comes into contact with an oxidising agent, such as the iron in haemoglobin. When mixed with hydrogen peroxide and a catalyst, the reaction excites the luminol molecules, which then release energy as visible blue light. Here, endogenous oxidants were harnessed to internally excite Ce6 through bioluminescence resonance energy transfer (BRET), decoupling therapy from external light.

The o/w single-emulsion method was used to obtain the Lum/Ce6-PLGA-NPs. An organic PLGA solution was added to an aqueous PVA solution. Then, an organic solution containing Ce6 and luminol (or Ce6 or luminol individually) was added, followed by sonication, stirring, and centrifugation. The particles were washed with water. The average diameter of Lum/Ce6-PLGA-NPs was approximately 120 nm. Bioluminescence of NPs was tested by mixing them with H_2_O_2_, myeloperoxidase (MPO), and/or ClO^−^. These compounds oxidised luminol, resulting in chemiluminescence of the NPs. The most sustained generation of luminescence was observed for samples containing all three components, specifically with hypochlorous acid (HClO) and hypochlorite (ClO^−^) anions. The ROS were generated by Ce6 via the bioluminescence resonance energy transfer (BRET) mechanism. Tests showed that ROS killed bacteria and promoted the apoptosis of neutrophils, reducing inflammation. The ROS generation assay revealed that the presence of H_2_O_2_ inhibited the generation process, which increased the selectivity of NP-based therapy. In vitro tests on *S. aureus* (3 × 10^4^ CFU) showed that a mix of Lum/Ce6-PLGA-NPs, H_2_O_2_, MPO, and Cl^−^ had a significantly stronger antibacterial effect than NPs alone and NPs containing either luminol or chlorin e6 individually. In vivo studies were conducted in mouse models of early-stage lipopolysaccharide (LPS)-induced inflammation, cecal ligation and puncture (CLP), *S. aureus*-induced peritonitis, vesicular stomatitis virus (VSV)-induced pneumonia, and bacterial-induced pneumonia. Lum/Ce6-PLGA-NPs in the treatment of bacterial-induced peritonitis accelerated bacterial clearance in major organs and induced neutrophil apoptosis, thereby downregulating the release of cytokines and protecting against infection. NPs in non-bacterial infections were also capable of reducing inflammation. The studies demonstrated a significantly higher percentage of surviving mice treated with Lum/Ce6-PLGA-NPs compared to those treated with a mix of Lum and Ce6 NPs or left untreated [71].

To evaluate the effect of drug hydrophobicity on NPs’ delivery, detailed research was conducted by Son et al. on PLGA NPs loaded with Ce6 (logD = −1.8) and pheophorbide *a* (Pba, Figure 9) with a logD of 4.01 [72]. The NPs were synthesised by first dissolving 1,2-dipalmitoyl-*sn*-glycero-3-phosphocholine (DPPC) and 1,2-distearoyl-*sn*-glycero-3-phosphoethanolamine with conjugated methoxyl poly(ethylene glycol) (DSPE-mPEG) in an alcohol-water solution. PLGA and Ce6 or Pba were dissolved in DMSO and added dropwise to the first solution, followed by sonic stirring to obtain nanoparticles stabilised by phospholipids. Unloaded photosensitizers were removed via dialysis in distilled water. DLS showed that Ce6-PLGA-NPs were larger (~160 nm), while Pba-PLGA-NPs were smaller (~120 nm). A drug release assay conducted in PBS revealed that Ce6-PLGA-NPs released the drug significantly faster than Pba-PLGA-NPs, likely due to the hydrophobic properties of Pba, whereaslight irradiation of the NPs, free Ce6, and free Pba demonstrated similar results in generating singlet oxygen. Cellular uptake assays were performed on SCC7 tumour-bearing mouse cells. Ce6-PLGA-NPs showed slightly better cellular uptake compared to free Ce6, whereas free Pba was absorbed significantly better by the cells than Pba-loaded NPs. To evaluate phototoxicity, MTT tests were conducted. No significant differences in cell viability were observed between the NP formulations and the free drug. In vivo tests conducted on SCC7 tumour-bearing mice revealed that Ce6-PLGA-NPs were slightly more specific in binding to tumours. Both Ce6 formulations bound to the tumour significantly faster than Pba, whether free or NP-loaded. Due to the enhanced permeability and retention effect, Pba-PLGA-NPs exhibited highly prolonged tumour accumulation compared to the free drug. PDT conducted on SCC7 tumour-bearing mice over 10 days demonstrated the significant superiority of Pba-PLGA-NPs (0.5 g of tumour mass after treatment) over Ce6-PLGA-NPs (1.5 g). In comparison, untreated tumours reached a mass of approximately 2.2 g. This research demonstrated that the hydrophobicity of the drug significantly influenced the efficacy of NP-based therapies. The authors concluded that the rapid release and low hydrophobicity of Ce6 may have contributed to weaker NPs accumulation at tumour sites, leading to incomplete therapeutic results. On the other hand, the prolonged interaction of highly hydrophobic Pba-PLGA-NPs resulted in effective suppression of tumour growth [72].

#### 2.3.3. Concluding Remarks on the Chlorin e6 Connection with Polymers

**Figure 10:**
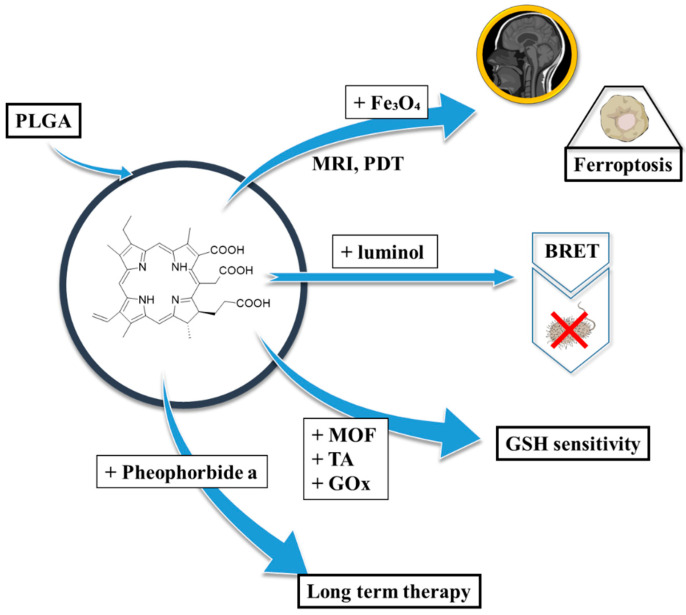
Chlorin e6 in polymeric nanoparticles—properties and applications; BRET—bioluminescence resonance energy transfer, GOx—glucose oxidase, GSH—glutathione, MOF—metal–organic framework, MRI—magnetic resonance imaging, PDT—photodynamic therapy, TA—tannic acid.

In conclusion, the encapsulation of chlorin e6 in PLGA-based nanoparticles represents a highly promising strategy to enhance the therapeutic efficacy and diagnostic versatility of photodynamic therapy. Across various formulations—ranging from PEGylated systems for improved stability and tumour accumulation, to multifunctional constructs incorporating magnetic nanoparticles, tannic acid, metal–organic frameworks, and synergistic agents like doxycycline and luminol (Figure 10)—Ce6-loaded PLGA NPs have demonstrated superior performance in cellular uptake, ROS generation, and tumour regression compared to free Ce6. These platforms have not only advanced tumour-targeted imaging and therapy but also shown potential in antimicrobial and anti-inflammatory applications. Importantly, the studies highlight the critical roles of NP composition, drug hydrophobicity, and surface modification in determining biodistribution, release profiles, and therapeutic outcomes.

### 2.4. Other Porphyrinoids in Connection with PLGA-Based Nanoparticles

**Figure 11:**
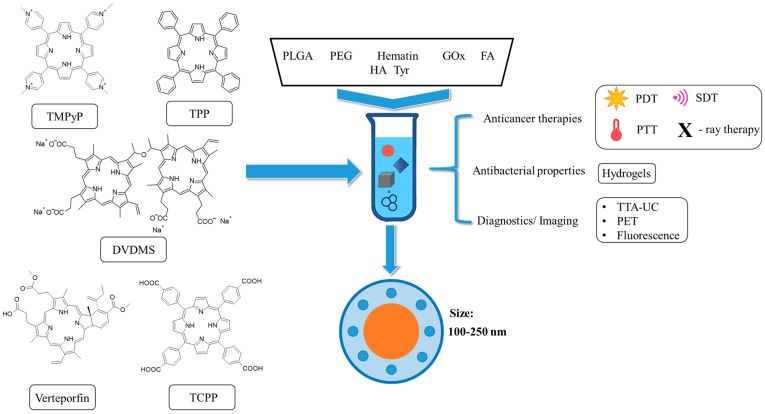
Different porphyrins in polymeric nanoparticles—properties and applications; FA—folic acid, GOx—glucose oxidase, HA—hyaluronic acid, PEG—polyethylene glycol, PET—positron emission tomography, PDT—photodynamic therapy, PTT—photothermal therapy, SDT—sonodynamic therapy, TTA-UC—triplet–triplet annihilation upconversion, Tyr—tyramine.

Over the past two decades, numerous porphyrin-based photosensitizers other than the above-mentioned porphyrin-based photosensitizers encapsulated in PLGA nanoparticles have been explored for their potential in photodynamic therapy and other biomedical applications. Their chemical structures are presented in Figure 11. One of the earlier comprehensive studies was conducted by Pegaz et al. in 2005, focusing on the influence of photosensitizer lipophilicity on extravasation behaviour and photocytotoxic efficacy [73,74]. The study included several porphyrins such as 5,10,15,20-tetrakis(4-hydroxyphenyl)porphyrin (pTHPP), 5,10,15,20-tetraphenylporphyrin (TPP), 5,10,15,20-tetrakis(4-carboxyphenyl)porphyrin (TCPP), Ce6, and Pba, all encapsulated in PLGA nanoparticles. Chlorin-loaded PLGA nanoparticles were prepared using the emulsification-diffusion method, whereas porphyrin-loaded systems employed the salting-out technique [51]. The resulting nanoparticles had an average size of approximately 200 nm, independent of the photosensitizer used. Polydispersity index values ranged from 0.046 to 0.323. Interestingly, encapsulation efficiency and drug loading were strongly influenced by the hydrophobicity of the compound—more hydrophobic photosensitizers generally exhibited lower EE. The authors proposed that lipophilicity governs not only the entrapment but also the release behaviour of the photosensitizers from the nanoparticles. In a chorioallantoic membrane (CAM) assay, it was observed that more hydrophilic dyes extravasated more rapidly, suggesting that hydrophobicity may limit diffusion from nanoparticles. Among the formulations tested, TPP-loaded nanoparticles demonstrated superior PDT efficacy compared to TCPP-loaded ones.

#### 2.4.1. Verteporfin in Connection with PLGA-Based Polymers

Further comparison with Verteporfin (VP, Visudyne^®^), a clinically approved photosensitizer for choroidal neovascularisation in age-related macular degeneration, revealed key differences in biodistribution. While VP rapidly extravasated from CAM vasculature, TPP-loaded PLGA nanoparticles remained more localised within blood vessels, potentially reducing collateral retinal damage [73].

VP has also been encapsulated in PLGA nanoparticles for enhanced delivery. Clement et al. aimed to overcome the limitations of visible light in treating deep-seated tumours by developing X-ray-activated PLGA-VP nanoparticles [75]. Nanoparticles were synthesised by a single emulsion-solvent evaporation method, yielding spherical particles of ~200 nm by TEM and ~250 nm by DLS, with PDI values between 0.03 and 0.09. To achieve targeted delivery, folic acid (FA) was conjugated to the PLGA-VP nanoparticles via amidation reaction between polymer and FA with the use of EDC/NHS ((1-ethyl-3-(3-dimethylaminopropyl)carbodiimide and N-hydroxysuccinimide) coupling. Targeting efficacy and phototoxicity were assessed in HCT116 (FR-overexpressing) and CCD841 CoN (normal colon epithelial) cells. While CCD841 cells remained unaffected, HCT116 cells showed a 67% reduction in viability under combined X-ray and nanoparticle treatment, highlighting the potential for selective deep-tumour PDT [75].

A study by Bazylinska and co-workers presented the co-encapsulation of cisplatin (CisPt) and VP into multifunctional PLGA-based nanocarriers stabilised by PLGA, PEG-PLGA, and FA-PLGA, using a double emulsion (w/o/w) technique [76]. Morphological analysis via TEM and AFM revealed smoother surfaces in pure PLGA shells and more defined core-shell structures in PEG/FA-modified systems. Nanoparticle sizes ranged from 187 to 200 nm, with PDI between 0.1 and 0.2. EE values were high, at ~95% for VP and 90% for CisPt. Cell uptake studies, conducted using flow cytometry and confocal microscopy, revealed an enhanced uptake of FA-functionalized nanoparticles, particularly for the VP+CisPt formulation. Photocytotoxicity studies on SKOV-3 and CHO-K1 cell lines showed a significant decrease in viability following longer incubation and light exposure, with minimal toxicity from empty nanoparticles, underscoring the protective effect of the PLGA matrix [76].

#### 2.4.2. Metal Tetraphenylporphyrins in Connection with PLGA-Based Polymers

To optimise nanoparticle formulation parameters, Mollaeva et al. proposed a two-level, three-factor design using an emulsion-solvent evaporation method. The study encapsulated three different metal *meso*-tetraphenylporphyrins (MePs)—Mn(III), Ni(II), and Co(II) porphyrins—into PLGA nanoparticles [77]. The MPs were synthesised according to established protocols [75] and characterised by ^1^H NMR spectroscopy. The optimisation variables included PLGA amount, PVA concentration, and the organic-to-aqueous phase ratio, with a Box–Behnken design (BBD) applied for statistical modelling. The resulting nanoparticles exhibited varying properties depending on the metal ion, with sizes of 322.9 nm (Ni), 344.5 nm (Co), and 205 nm (Mn); and zeta potentials of −14.7 mV, −10.7 mV, and +18.1 mV, respectively. Drug loading and EE were also variable, with MnClTPP (manganese(III)-tetraphenylporphyrin chloride) showing the highest values (28.9% and 79.9%, respectively). Their characterisation by TEM confirmed a core-shell morphology, while XRD analysis indicated crystallinity. FTIR spectra revealed no chemical interactions between PLGA and MfPs. Haemolysis tests demonstrated good biocompatibility, with MnClTPP-NPs showing minimal haemolysis (1.8%) even at low concentrations. Release studies indicated a biphasic release pattern governed by Fickian diffusion. In vitro cytotoxicity assays against HeLa, MCF-7, and SK-OV-3 cells showed that MePs and MeP-NPs alone had weak cytotoxic effects, whereas formulations combined with ascorbic acid exhibited significantly enhanced activity. Acute toxicity studies demonstrated safe dosing up to 200 mg/kg. In vivo pharmacokinetics revealed that MnClTPP and CoTPP (cobalt(II)-tetraphenylporphirin)-loaded nanoparticles resulted in lower plasma concentrations than their free forms, indicating altered biodistribution, while NiTPP (nickel(II)-tetraphenylporphyrin) showed no significant difference [77].

#### 2.4.3. Hematin in Connection with PLGA-Based Polymers

Another noteworthy approach was reported by Amin et al., who developed PLGA nanoparticles conjugated with hematin, an iron-containing blood porphyrin [78]. Unlike encapsulation, surface conjugation was used herein to decorate the carrier with a bioactive porphyrin, potentially altering cell interactions. The nanoparticles were synthesised via a modified emulsion-solvent evaporation technique. After the formation of PLGA nanoparticles, surface modification was achieved using L-arginine through EDC/NHS coupling, enabling conjugation with hematin. Excess unconjugated hematin was removed post-reaction. Characterisation by SEM and AFM revealed uniform, spherical particles with smooth surfaces. FTIR spectra confirmed successful surface functionalization and hematin conjugation. DLS analysis revealed an increase in particle size with each modification step (from 99.6 ± 8.21 nm to 127.5 ± 9.93 nm), while the zeta potential shifted from negative to positive and back to negative values, indicating changes in surface chemistry. The cellular uptake study, performed on HeLa cells, demonstrated significantly enhanced internalisation of hematin-functionalized nanoparticles compared to controls, suggesting that surface modification facilitated efficient cellular association [78].

#### 2.4.4. TMPyP in Connection with PLGA-Based Polymers

In 2016, a research group from Portugal introduced a novel approach, in which they employed TMPyP (5,10,15,20-tetrakis(1-methylpyridinium-4-yl)-porphyrin tetra-iodide) as a photosensitizer encapsulated within PLGA nanoparticles for topical antimicrobial therapy, aiming to address bacterial resistance to antibiotics [79]. Topical hydrogels were used as a secondary matrix to retain NPs on the tissue and sustain release at the infection site. The study utilised Carbopol hydrogels containing both free TMPyP and TMPyP-PLGA formulations to overcome limitations related to tissue uptake, drug retention, and solubility. TMPyP was synthesised based on established protocols [80], and both unloaded and TMPyP-loaded PLGA nanoparticles were produced using the solvent evaporation technique. These nanoparticles exhibited comparable sizes, with an average diameter of 117 nm for empty PLGA and a range of 118 nm to 133 nm for TMPyP-loaded variants, depending on the photosensitizer concentration. All formulations demonstrated a negative surface charge, with zeta potentials from −21.6 mV to −26.7 mV, and polydispersity indices (PDI) of 0.14 for void PLGA and approximately 0.17 for TMPyP-PLGA. The nanoparticle morphology was examined via SEM, and the encapsulation efficiency was quantified. Hydrogels were prepared by incorporating the nanoparticles into a Carbopol 940 aqueous solution, followed by the addition of propylene glycol, ethanol, and triethanolamine. These formulations showed only a slight decrease in viscosity after six months, while pH remained stable. The hydrogels were characterised using UV-Vis absorption spectroscopy, singlet oxygen generation assays, and photobleaching studies. Both free and encapsulated TMPyP hydrogels generated sufficient singlet oxygen, with superior photostability observed in the PLGA-encapsulated form. Drug release kinetics were studied fluorometrically after suspending the hydrogels in the release medium, showing moderate release of TMPyP. Franz cell diffusion studies using intact porcine skin indicated effective penetration and biocompatibility, supporting their potential application in photodynamic inactivation (PDI) for antimicrobial treatment or PDT [79].

A more recent development in 2024 by Chi et al. explored microneedle (MN) technology as a refined platform for TMPyP delivery [81]. Despite the advantages of MNs in transdermal drug delivery, challenges persist in achieving optimal mechanical properties and delivery efficiency. To address these, enzyme-mediated cross-linking of hydrogel polymers, particularly hyaluronic acid (HA), was employed. The researchers developed a formulation wherein PLGA-encapsulated TMPyP nanoparticles were embedded in HA-tyramine (HAT) hydrogels, resulting in a composite denoted as HAT@NP/TMPyP. These nanoparticles were synthesised via a water-in-oil-in-water double emulsion technique, achieving a loading capacity of 6.2% and an encapsulation efficiency of 59.7%. Transmission electron microscopy confirmed spherical morphology with a size around 100 nm, consistent with DLS results of 117 nm and a low PDI of 0.066. HA-Tyr conjugates were synthesised through EDC/NHS coupling and characterised using ^1^H NMR and UV-Vis spectroscopy. The obtained hydrogels were then used to fabricate dissolvable MN patches. The resulting MNs demonstrated uniform morphology, minimal leakage of TMPyP, high room-temperature stability, and sufficient mechanical strength for skin insertion. Transdermal delivery studies in porcine skin and retention time assessments in murine models indicated that HAT-Medium@NP/TMPyP provided optimal delivery and sustained presence. In vitro cytotoxicity assays against A375 human melanoma cells demonstrated superior photocytotoxic effects for this formulation (IC_50_ = 17 µg/mL). Furthermore, in vivo treatment in A375 xenografted mice with HAT-Medium@NP/TMPyP and light exposure resulted in pronounced tumour regression and elevated apoptotic cell counts relative to controls [81].

In a separate study published in 2019, Ito and co-workers developed a new therapeutic delivery system utilising manganese porphyrin (MnTMPyP) encapsulated in PLGA nanospheres [82]. MnTM4PyP, a quaternary chloride salt of manganese(III) meso-tetrakis(1-methylpyridinium-4-yl) porphyrin, was synthesised following established methods [83] and characterised using UV-Vis spectroscopy. Nanospheres were prepared via a w/o/w emulsion method, and exhibited zeta potentials between −9 mV and −14 mV. A parametric study was conducted to investigate the impact of PLGA concentration, phase ratios, surfactants, and polymer molecular weight on particle size and loading efficiency. An increase in PLGA quantity correlated with larger particle sizes, while molecular weight and phase volume ratios influenced the drug loading. Release profiles indicated that lower molecular weight PLGA yielded more efficient release, demonstrating that drug delivery characteristics could be fine-tuned through formulation variables [82].

In 2014, Laster et al. utilised a palladium(II) complex of TMPyP, PdTMPyP4, encapsulated in PLGA as a means to inhibit telomere elongation in cancer cells [84]. Instead of PDT, the porphyrin serves here as a telomerase-targeting agent, with PLGA rods enabling in situ, months-scale release. This formulation, produced as solid rods via a patented method, was intended for direct tumour implantation. In vitro studies showed linear drug release over 48–52 days, correlating with rod disintegration, but independent of rod mass. Ex vivo analysis post-implantation indicated continued release of PdTMPyP4. Cellular uptake studies using ICP-MS demonstrated accumulation of over 10^9^ Pd atoms per DNA molecule in L-428 Hodgkin’s lymphoma cells. Telomerase activity, measured by TRAP assay, declined by ~15% within 24 h of exposure. Long-term exposure, spanning 11 to 19 days, resulted in reduced cell survival, with an observed latency period of ~8 days between telomerase inhibition and telomere shortening. In vivo testing in a KHJJ adenocarcinoma BALB/c mouse model revealed tumour growth retardation by a factor of 3–5 compared to controls, indicating therapeutic potential in limiting tumour proliferation without affecting healthy tissues [84].

#### 2.4.5. TCPP in Connection with PLGA-Based Polymers

Investigating another porphyrin, Hu et al. in 2009 encapsulated meso-tetra(carboxyphenyl)porphyrin (TCPP, Figure 11) into PLGA nanoparticles for PDT applications [85]. TCPP was synthesised according to previous methods [86] and encapsulated using a single-emulsion solvent evaporation method, yielding nanoparticles with an average diameter of 65 nm and a narrow size distribution (50–70 nm, as determined by SEM). Cellular uptake studies in SW480, LS174T, and HT-29 colon cancer cell lines indicated rapid internalisation of both free and encapsulated TCPP, with maximum uptake at 3 h. J774 macrophages exhibited lower uptake of TCPP NPs. Fluorescence imaging and molecular assays (Western blot, RT-PCR) confirmed clathrin-mediated endocytosis as the predominant internalisation mechanism. MTT assays showed that TCPP NPs had the highest photocytotoxicity among the tested formulations. In vivo studies on SW480 xenografts in athymic mice demonstrated significant tumour growth delay post-TCPP NP injection [85].

To address limitations in photothermal therapy (PTT), Zhang and co-workers, in a 2021 study, developed PEG-b-PLGA nanoparticles co-loaded with TCPP and the anticancer agent isolienisinine (Iso) for combined PTT and chemotherapy [87]. Nanoparticles were prepared using anti-solvent precipitation and exhibited spherical morphology with sizes ranging from 75 to 108 nm and PDI between 0.2 and 0.4. Encapsulation efficiencies were 18% for Iso and 16% for TCPP. FT-IR and UV-Vis spectroscopy confirmed successful encapsulation. Approximately 58% of TCPP and 42% of Iso were released within 12 h. Upon light irradiation, the temperature increased proportionally with TCPP concentration. Cytotoxicity tests on NIH 3T3 and MDA-MB-231 cell lines revealed minimal toxicity to normal cells, while tumour cells showed 9% viability at 30 µg/mL, confirming the synergistic therapeutic effect [87].

#### 2.4.6. Several Further Selected Porphyrins in Connection with PLGA-Based Polymers

A different strategy was introduced by Bruno in 2015 to enhance cytosolic antigen delivery for CD8 T cell activation via PLGA microparticles co-loaded with ovalbumin (OVA) and the photosensitizer TPCS2a (tetraphenyl chlorine disulphonate) [88]. These were prepared via a w/o/w double emulsion method, with microparticles ranging from 0.4 to 1.3 µm in size (2−3.5 µm post-lyophilisation) and zeta potentials from −20 to −12 mV. Encapsulation efficiencies were 69% for OVA alone and 58% for OVA-TPCS2a co-formulations. In vivo photosensitization studies in C57BL/6 mice that received Rag2/OT-I lymphocytes demonstrated enhanced CD8 T cell responses and increased production of IFN-γ, TNF-α, and IL-2 following administration of PLGA-OVA-TPCS2a. Cytotoxicity assays revealed effective cell killing via granzyme B activity. This delivery system induced MHC I-predominant responses, suggesting its potential for use in vaccines targeting CD8 T cell-mediated diseases [88].

While cell-based in vitro models, such as 2D or 3D cell cultures, provide an initial assessment of drug efficacy, they are limited in their ability to predict in vivo outcomes. In 2022, Elberskirch and colleagues proposed a new tumour model for anticancer drug screening based on the Hen’s egg test on the chorioallantoic membrane (HET-CAM) model [89]. Two distinct carriers were formulated for biological assays: PLGA nanoparticles with Temoporfin as PS and liposomes containing two novel photosensitizers (LC 2175 and BLC 5152) synthesised according to established methods [90], involving nucleophilic aromatic substitution on 5,10,15,20-tetrakis(pentafluorophenyl)porphyrin with two amines. Nanoparticles with muco-adhesive and permeating properties were produced via the solvent evaporation method: PLGA-Carbopol^®^ (CP) (NP-PLGA-mTHPC (Temoporfin)-CP) [89]—and via w/o/w double emulsion solvent evaporation—PLGA-Poloxamer 407 (NP-PLGA-mTHPC-F127) [91]. The resulting nanoparticles had the diameters of 124.1 nm (PDI: 0.03, zeta potential: –52.8 mV) and 115.4 nm (PDI: 0.09, zeta potential: –46.5 mV), respectively. Tumour spheroids were developed from the adenocarcinoma cell line HuTu-80 and the human colon cancer cell line HT29-MTX-E12, following a previously described protocol [89]. These spheroids were applied to the CAM, with an approximate success rate of 70%, and were treated with both free and nanoparticulate photosensitizers via pipetting onto the spheroid surface. The accumulation of LP-BLC2175, LP-BLC5152, and NP-PLGA-mTHPC-CP was comparable to that of mTHPC, while NP-PLGA-mTHPC-F127 showed significantly higher accumulation. Increased accumulation was noted between 24 and 48 h. None of the tested photosensitizers or nanoformulations exhibited dark toxicity, while all showed phototoxicity upon irradiation, except controls. The highest photocytotoxicity was observed for mTHPC and NP-PLGA-mTHPC-F127. Quantification of viable, necrotic, and apoptotic cells in the spheroids was also achieved [89].

In 2019, Galliani and Signore proposed a method to enhance photodynamic therapy by using quantum dots (QDs) to excite photosensitizers via a FRET-based mechanism, aiming to address poor solubility, aggregation, and insufficient clearance of traditional photosensitizers [92]. Co-encapsulating QDs with a porphyrinoid creates an internal light source/transducer to drive PDT at otherwise suboptimal wavelengths. The authors encapsulated a chlorophyllin copper complex (Chl) and CdSe/ZnS core-shell QDs into PLGA nanoparticles using a modified nanoprecipitation method. The resulting Chl-QD-loaded NPs had hydrodynamic diameters ranging from 169 to 220 nm and a zeta potential of approximately –30 mV, suggesting that the encapsulants had a minimal effect on nanoparticle formation. The co-encapsulation significantly increased EE from 4% to 20% for Chl and from 50% to 90% for QDs. Photocytotoxicity testing on NIH-2T2 cells revealed that Chl QD NPs were twice as effective under UV light as in the dark, and more effective than any other formulation, confirming the improved therapeutic potential of the co-formulated system.

In 2020, Mai and co-workers developed a hydrogel-based nanodelivery system for antibacterial action and skin regeneration in burn wound treatment, using photodynamic antimicrobial chemotherapy (PACT) [93]. Fibroblast growth factor (bFGF) was encapsulated into PLGA nanoparticles via the double emulsion–solvent evaporation method [94], producing uniform spherical nanoparticles (diameter: 415 nm, zeta potential: –9.86 mV). These nanoparticles were embedded in the CSDP (carboxymethyl chitosan—sodium alginate) hybrid hydrogel alongside sinoporphyrin sodium (DVDMS, Figure 11). The composite hydrogel released bFGF over 18 days and exhibited stretchability (125.13–142.37%) exceeding that of native skin. CSDP hydrogels also had superior elasticity and fatigue resistance compared to silk-based hydrogels. Antibacterial assays against *S. aureus* and MDR-*S. aureus* demonstrated enhanced efficacy, while PLGA-bFGF alone had no antibacterial effect. In a haemorrhaging liver model, CSDP hydrogel significantly reduced blood loss. The hydrogel showed no haemolytic or skin toxicity and achieved 99.99% bacterial eradication under mild photoirradiation. In vivo, the formulation enabled sterilisation and rapid wound healing in burn models [93].

To support real-time monitoring of tissue oxygenation in vivo, Presley et al. incorporated Pd(II) benzoporphyrin into polymer matrices (PCL, PCL:gelatin, and PCL:PLGA blends or core-shells) using electrospinning [95]. SEM images revealed that PCL and PCL:gelatin-PCL core-shells had wrinkled morphologies, with the latter exhibiting a combination of cylindrical and flattened fibres. PCL:PLGA fibres appeared smoother. Fibre diameters ranged from 0.31 to 0.72 µm. Phosphorescence and excitation spectra showed no signs of porphyrin aggregation. A custom in situ fluorimeter setup revealed high oxygen sensitivity in PCL fibres (KSV: 1.25 × 10^5^ M^−1^) with monoexponential decay, indicating a homogeneous chromophore environment. PCL:gelatin also displayed promising sensitivity but with reduced stability. The PCL shell improved sensor longevity. However, PCL:PLGA blends exhibited poor sensitivity and heterogeneity, rendering them unsuitable for further study. Degradation testing confirmed PCL:gelatin’s faster degradation and the intermediate rate of PCL:gelatin-PCL [95].

In the field of boron neutron capture therapy (BNCT), Shi and co-workers developed PLGA-mPEG nanoparticles (boronated porphyrin nanocomplex, BPNs) encapsulating porphyrins for fluorescence and PET imaging, as well as boron delivery [96]. PLGA-mPEG was synthesised via ring-opening polymerisation, and the BPNs were produced via dialysis [97]. TEM images confirmed monodisperse, spherical micelles (~100 nm, PDI: 0.1, zeta potential: –39 mV). Cytotoxicity tests revealed high toxicity for the boronated porphyrin, moderate for BPNs, and low for PLGA-mPEG, with 77% cell viability in BPN-treated B16-F10 cells. Boron uptake (250 ppm after 48 h) was confirmed via ICP-OES and microscopy. BPNs demonstrated strong fluorescence for optical imaging and were effectively labelled with Cu-64 for PET imaging. Tumour accumulation was high, and excretion occurred via the hepatobiliary route. Repeated dosing improved boron delivery efficiency, and BPNs suppressed tumour growth post-BNCT with minimal side effects [96].

In 2022, Vepris and co-workers designed PLGA nanoparticles for triplet–triplet annihilation upconversion (TTA-UC) imaging using PtOEP (platinum(II) octaethylporphyrin) and 9,10-diphenylanthracene (DPA) [98]. These nanoparticles were synthesised using solvent evaporation, resulting in particles sized 200 nm (PDI: 0.4, zeta potential: –31 mV). The EE for PtOEP and DPA was 24.4% and 39.6%, respectively. No cytotoxicity was observed in OVCAR-3 cells after 72 h. Cellular uptake was confirmed by fluorescent microscopy, and in vivo studies in mice demonstrated tumour accumulation from 3 to 96 h post-injection, highlighting the system’s potential for cancer imaging.

Last but not least, in 2021, Wang et al. reported a theranostic nanoparticle system for combined sonodynamic therapy (SDT) and starvation therapy using PLGA nanoparticles encapsulating glucose oxidase (GOx) and manganese(III) 5,10,15,20-tetrakis (4-chlorophenyl)porphyrin) chloride, denoted as PMnC), a porphyrin sonosensitizer [99]. By co-packaging a metabolic enzyme with a sonosensitizer, the platform attacks tumours via nutrient deprivation and ultrasound-activated ROS. The MG@P nanoparticles, produced by double emulsion, had a diameter of 278.3 nm, zeta potential of –26.7 mV, and high EE for PMnC (95.83%) and GOx (23.80%). Catalytic activity was preserved post-encapsulation. Flow cytometry revealed high ROS production in 4T1 cells under ultrasound exposure. CCK-8 assay showed synergistic cytotoxicity from combined therapies. In vivo, MG@P NPs exhibited peak imaging signal at 24 h and strong tumour growth inhibition, with biosafety studies indicating reversible effects on blood glucose and no organ toxicity. The formulation offers a multifunctional platform for SDT, starvation therapy, and imaging [99].

#### 2.4.7. Concluding Remarks

From improving the in vivo predictability of anticancer drug screening in HET-CAM tumour models to enhancing treatment efficacy through photodynamic, photothermal, sonodynamic, and boron neutron capture therapies, the integration of porphyrins with nanocarriers has led to notable gains in targeting, bioavailability, and multifunctionality. Innovations such as upconversion imaging, quantum dot co-encapsulation, hydrogel composites, and multimodal theranostics highlight the ongoing efforts to create safer, more efficient, and tunable systems for cancer therapy and tissue regeneration (Figure 11).

## 3. The Analysis of Particle Size, Zeta Potential and Encapsulation Efficiencies of Selected Porphyrinoids in PLGA

**Figure 12:**
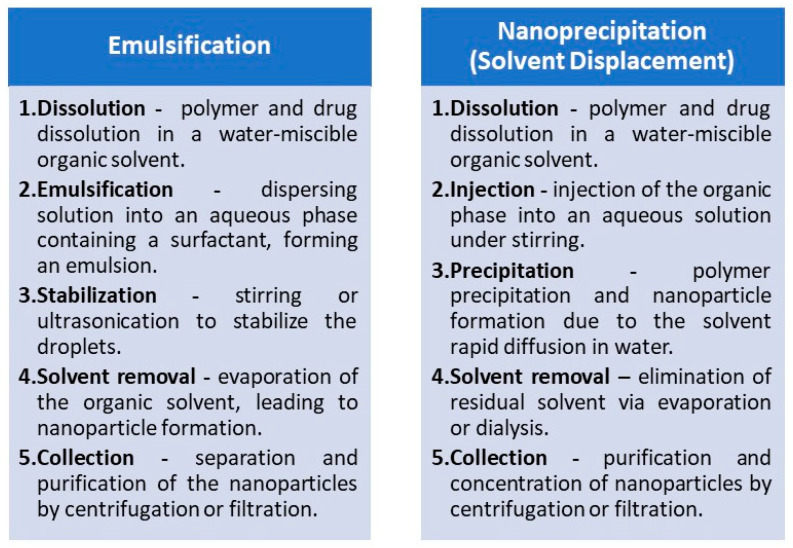
The steps used in the preparation of polymeric drug delivery systems by emulsification and nanoprecipitation methods.

Based on the data provided by the authors of the reviewed studies, we were able to compare parameters such as particle size, zeta potential, polydispersity index (PDI), and encapsulation efficiency (EE), where available. We deliberately excluded the data presented in subchapter 2.4 concerning other types of PLGA-encapsulated porphyrins, as a sufficient number of studies were conducted only for PPIX, THPP, and Ce6. Methods such as emulsification and nanoprecipitation are commonly used to fabricate these carriers (Figure 12) [17].

The analysis of blank PLGA nanoparticles, which are commonly used as controls, served as a starting point for consideration. The physicochemical characterisation of unloaded PLGA nanoparticles across multiple studies shows considerable variation in the aforementioned parameters, reflecting differences in formulation protocols and experimental conditions (Table 1). Reported particle sizes ranged from 99.6 nm [78] to 241 nm [75], with most values falling within the optimal nanometric range for drug delivery applications (<150 nm) [100]. Zeta potential values, where available, varied between −11.9 mV and −38 mV, indicating a generally negative surface charge that contributes to colloidal stability. The most negative zeta potential was observed in da Silva’s work [33], suggesting improved repulsion between particles and potentially enhanced dispersion stability. PDI values across studies remained low (0.05–0.2), with most values below 0.1, indicating a narrow particle size distribution and homogeneity. The calculated mean values were as follows: mean particle size of 161.8 nm, a mean zeta potential of −24.5 mV, and a PDI of 0.09.

The data concerning PPIX encapsulation is presented in Table 2. In the analysed studies, particle sizes ranged from 90 nm [44] to 290 nm [33], generally falling within a favourable range for passive targeting in drug delivery. A trend was observed where more recent formulations, such as those by Dinakaran et al. [44] and Azad et al. [45], achieved significantly smaller particle sizes (<100 nm), potentially enhancing cellular uptake and biodistribution. Zeta potential values, when reported, were consistently negative, varying from −12.2 mV to −32.3 mV, indicating stable colloidal suspensions. PDI values were moderate (0.162–0.233), suggesting acceptable size uniformity, though slightly higher than those of empty nanoparticles, likely due to the inclusion of PPIX. The mean size of PLGA/PPIX nanoparticles, measured by DLS, was 193.3 nm, which is an acceptable size for the biological studies, while when the desired particle is not a nanoparticle, but a microsphere or nanofibers, the size, measured from microscope photos, is much larger: 2 µm to 150 µm, respectively. The mean zeta potential was −22.6 mV (as calculated from the provided data), and the mean PDI was 0.208, indicating low polydispersity; however, only four papers provided a polydispersity index. Encapsulation efficiency varied widely, from as low as 6.24% [42] to as high as 90% [44], with higher efficiency typically associated with smaller particle sizes. According to the literature data, the encapsulation efficiency of photosensitizers in PLGA nanoparticles stems from the initial concentration of PS, where a lower concentration results in a higher PS-polymer affinity, contributing to the higher EE observed [101,102].

Regarding other studies, THPP-loaded PLGA nanoparticles exhibited substantial variations in particle size, surface charge, and encapsulation efficiency, depending on the formulation strategies and experimental conditions (Table 3). Particle sizes ranged from as low as 70.4 nm [57] to as high as 350 nm [58], with most formulations remaining under 120 nm, which is generally considered favourable for enhanced cellular uptake and tumour penetration [100]. The mean particle size was 175.2 nm. Zeta potential values were predominantly negative, spanning from −5.25 mV [50] to −45.9 mV [56], suggesting differing surface stabilisation depending on process parameters. PDI values, when reported, were low (0.04–0.25), indicating monodisperse nanoparticle populations in most cases, although the formulation obtained by Forouharshad and co-workers [58] showed a broader distribution. Encapsulation efficiency varied significantly, with high values observed in recent studies (81.01–88.91%), indicating an improvement in loading techniques over time. Notably, formulations with smaller particle sizes and more negative zeta potentials generally corresponded to higher encapsulation efficiencies, implying that increased surface area and particle stability may enhance loading capacity [103].

In the case of Ce6, the reported particle sizes of the Ce6-loaded PLGA nanoparticles measured using DLS ranged from 100 nm [67] to 531.17 nm [70] (mean size of 229.65 nm) and for the majority of formulations maintaining sizes below 250 nm, suitable for passive targeting and enhanced permeation and retention (EPR) effects (Table 4). The zeta potential values were consistently negative, ranging from −18.57 mV to −38.4 mV, suggesting good colloidal stability across all systems. PDI values, when reported, were exceptionally low (0.036–0.086), indicating highly uniform particle populations. Only a subset of studies reported encapsulation efficiency, with FTCPNPs [68] showing a high value of 82.09%, while Liang et al. [70] reported a relatively modest 46.16% for a larger-sized formulation. Notably, the presence of inorganic components such as Fe_3_O_4_ or coordination complexes (e.g., Fe^3+^-tannic acid) appeared to influence both particle size and surface charge, indicating that Ce6 encapsulation within PLGA can be finely tuned through co-loading or surface functionalization strategies.

Finally, regarding the photosensitizers described in Section 2.4, the PLGA nanoparticle method of preparation, particle size, zeta potential, PDI, and EE values are summarised in Table 5. The particle sizes measured with the use of DLS ranged from 87 nm [87] to 415 nm [93], whereas zeta potentials spanned between −52.8 mV [79] and +18.1 mV [77]. However, due to noticeable differences in the chemical structures of encapsulated photosensitizers, a direct comparison of each material cannot be performed, as the PSs’ structure can affect the PS-polymer affinity and, therefore, alter the morphology and physical properties of the nanoparticles.

## 4. Conclusions and Perspectives

Porphyrinoid-type photosensitizers encapsulated in PLGA nanoparticles have been widely investigated for the last 20 years as a promising strategy for enhancing photodynamic therapy. Compared with free photosensitizers, PLGA-based nanocarriers improve solubility, stability, and circulation time, while protecting the photosensitizer from premature degradation or aggregation. These advantages stem from the tunable structure and characteristics of PLGA, such as different monomer ratios, which can adjust its hydrophobicity and degradation rate. As a result, PLGA directly influences the loading, release rate, and behaviour of photosensitizers in biological environments. Studies across different porphyrinoids consistently demonstrate that encapsulation modifies physicochemical parameters such as particle size, surface charge, and release kinetics, which in turn determine cellular uptake, subcellular localisation, and PDT efficacy. In many cases, PLGA-based formulations outperform free drugs in terms of accumulation in the tumour, retention, and therapeutic selectivity.

The reviewed findings highlight several trends. Firstly, the balance between hydrophobicity and hydrophilicity of photosensitizers strongly influences encapsulation efficiency, loading capacity, and release behaviour. Secondly, the polymer composition (lactic-to-glycolic acid ratio and molecular weight) as well as the formulation method determines the stability and biodistribution of nanoparticles. Thirdly, surface functionalization, achieved through PEGylation, chitosan modification, or lipid-hybrid coatings, provides additional opportunities to increase stability, enhance mucus penetration, and improve active or passive targeting. Lastly, multifunctional designs enable the integration of imaging agents or therapeutic co-drugs, allowing for combined modalities such as chemo-photodynamic therapy, photothermal-photodynamic therapy, and even ferroptosis-based approaches.

Despite these advances, several challenges have to be addressed before PLGA-based porphyrinoid systems are translated into clinical practice. One limitation lies in the scale-up and the ability to consistently reproduce the nanoparticle synthesis, as small variations in emulsification or solvent diffusion protocols can noticeably alter particle characteristics. Another major issue is premature release or rapid efflux of photosensitizers, which may reduce therapeutic selectivity. Strategies such as PEGylation, hybrid lipid shells, and stereocomplex stabilisation have been proposed, but further refinement is needed before these can be seriously considered. Additionally, biodistribution studies have frequently shown significant accumulation in the liver and spleen, raising concerns about off-target effects and potential clearance pathways.

Future perspectives in this field should address three main directions. First, systematic comparisons of different types of porphyrinoids in standardised PLGA formulations are required to establish structure-activity relationships and identify the most promising candidates for specific clinical applications. Second, integration of stimuli-responsive elements, such as pH-sensitive linkers, redox-triggered release, or ultrasound/near-infrared activation, should be pursued to further enhance selectivity and control drug release. Third, translation to clinically relevant models must be prioritised. While most current evidence is derived from in vitro assays or small animal models, large-scale preclinical studies are needed to evaluate pharmacokinetics, immune interactions, and long-term safety.

## Figures and Tables

**Table 1:** The physical parameters of blank PLGA nanoparticles.

Preparation Method	LA to GA Ratio	Size [nm]	Zeta Potential [mV]	PDI	Ref.
nanoprecipitation	50:50	176.6 ± 17.55	−38.0 ± 7.41	0.056	[33]
nanoprecipitation	50:50	200.0 ± 20	Not specified	0.05 ± 0.004	[34]
nanoprecipitation	50:50	200.0 ± 20	Not specified	0.05	[35]
single-emulsion-solvent evaporation	50:50	116.3 ± 36.7	−11.9 ± 0.6	-	[36]
single-emulsion-solvent evaporation	50:50	241 ± 4	−20 ± 1	0.07 ± 0.03	[75]
emulsion-solvent evaporation	50:50	99.6 ± 8.21	−25.31 ± 1.04	-	[78]
evaporation method	50:50 and 75:25	117 ± 5 to 126 ± 7	From −26.7 ± 1.5 to −25.9 ± 1.2	0.08 ± 0.02 to 0.14 ± 0.02	[79]
Not specified	50:50	180 ± 80	−24 ± 6	0.2	[98]

**Table 2:** The physical parameters of PPIX/PLGA nanoparticles.

Name	Type of NP	LA to GA Ratio	Size [nm]		Zeta Potential [mV]	PDI	EE	Ref.
	Preparation Method		DLS	TEM/SEM				
PPIX/PLGA	nanoparticles	50:50	290.0 ± 67.21	-	−32.3 ± 8.16	0.233	67.7%	[33]
	nanoprecipitation							
PPIX/PLGA	nanoparticles	50:50	280 ± 60.2	-	-	0.22 ± 0.1	68.0 ± 12.0%	[34]
	nanoprecipitation							
PPIX/PLGA	nanoparticles	50:50	278 ± 60	-	-	0.22	-	[35]
	nanoprecipitation							
PPIX/HA-b-PLGA	core-shell micelles	50:50	213.4	150	−24.3	0.152	42.9 ± 2.0%	[37]
	solvent dialysis							
PPIX/PLGA	nanoparticles	50:50	320	-	-	-	-	
	nanoprecipitation							
GT-PLGA	shell-core nanofibers	NS	-	110–150 µm	-	-	-	[41]
	coaxial electrospinning technol.							
PPIX/GT-CS/PLGA	shell-core nanofibers	NS	-	110–150 µm	-	-	-	
	coaxial electrospinning technol.							
PPIX/GT-HA/PLGA	shell-core nanofibers	NS	-	110–150 µm	-	-	-	
	coaxial electrospinning technol.							
PPIX/PLGA	nanoparticles	50:50	121.5 ± 44.5	33.6 ± 9	−12.2 ± 1	-	13.7 ± 1.7 wt.%	[36]
	single-emulsion-solvent evapor.							
UCNPs/PPIX/PLGA-PEG	nanoparticles	NS	-	288.1 ± 49.2	-	-	6.24 ± 0.8 wt.%	[42]
	nanoprecipitation							
LaF_3_:Ce^3+^/PPIX/PLGA	microspheres	NS	-	approx. 2 µm	-	-	-	[43]
	emulsion/evaporation technique							
LaF_3_:Ce^3+^(NSCs)/PPIX/PEG-PLGA	core-shell structures	NS	90 to 120	-	−15 to −30	<0.3	90%	[44]
	nanoprecipitation							
PPIX/NSCs/PEG-PLGA	nanoparticles	NS	96 ± 6	-	−27.4 ± 0.5	<0.25	-	[45]
rac-[Ru(phen)_2_(IP-4T)](Cl)_2_/NSCs/PEG-PLGA	nanoparticles	NS	118 ± 3	-	−17.4 ± 0.7	<0.25	-	

Abbreviations: NS—not specified.

**Table 3:** The physical parameters of THPP/PLGA nanoparticles.

Name	Type of NP	LA to GA Ratio	Size [nm]	Zeta Potential [mV]	PDI	EE	Ref.
	Preparation Method						
pTHPP/PLGA (50:50)	nanoparticles	50:50	117 ± 7	-	0.20	76.9 ± 3.4%	[50]
	emulsification-diffusion						
pTHPP/PLGA (75:25)	nanoparticles	75:25	118 ± 2	-	0.20	77.0 ± 7.0%	
	emulsification-diffusion						
pTHPP/PLA	nanoparticles	-	125 ± 1	-	0.16	72.8 ± 8.7%	
	emulsification-diffusion						
pTHPP/PLGA (50:50)	nanoparticles	50:50	93 ± 0–145 ± 1	−5.8 ± 0.3 to −4.7 ± 1.4	-	56.2 ± 10.5–76.9 ± 3.4%	[51]
	emulsification-diffusion						
pTHPP/PLGA (75:25)	nanoparticles	75:25	95 ± 6–157 ± 7	−6.6 ± 1.9 to −4.2 ± 0.7	-	47.0 ± 3.2–77.4 ± 7.0%	
	emulsification-diffusion						
pTHPP/PLA	nanoparticles	-	104 ± 1–134 ± 6	−7.8 ± 1.1 to −4.3 ± 0.8	-	61.2 ± 0.5–91.1 ± 6.4%	
	emulsification-diffusion						
pTHPP/PLGA	nanoparticles	50:50	117 ± 7	-	0.2		[53]
	emulsification-diffusion						
mTHPP/PLGA (6% PVA)	nanoparticles	50:50	593 ± 15	-	0.08		[54]
	emulsification-diffusion						
mTHPP/PLGA (9% PVA)	nanoparticles	50:50	285 ± 7	-	0.05		
	emulsification-diffusion						
mTHPP/PLGA (17% PVA)	nanoparticles	50:50	117 ± 8	-	0.04		
	emulsification-diffusion						
mTHPP/PLGA	nanoparticles	50:50	245.6 ± 9.9	−41.8 ± 3.2	0.07 ± 0.01		[55]
	emulsification-diffusion						
mTHPP/PLA	nanoparticles	-	285.6 ± 15.3	−37.1 ± 5.6	0.16 ± 0.05		
	emulsification-diffusion						
mTHPP/ Eudragit-E^®^	nanoparticles	-	213.6 ± 5.6	+55.0 ± 3.4	0.06 ± 0.02		
	emulsification-diffusion						
pTHPP/PLGA	core-shell nanoparticles	50:50	88.5 ± 3.4–94.6 ± 2.7	−43.2 ± 1.6 to −48.6 ± 1.9	0.06 to 0.11	73.12 ± 3.17–88.91 ± 2.07%	[56]
	nanoprecipitation						
pTHPP/PHBV	core-shell nanoparticles	-	213.7 ± 4.0–230 ± 5.9	−35.4 ± 2.5 to −39.7 ± 1.6	0.10 to 0.14	67.47 ± 3.61–77.91 ± 3.83%	
	nanoprecipitation						
pTHPP/PLHNPs	PLGA-lipid hybrid NPs	50:50	70.4 ± 1.4	−39.2 ± 0.8	-	88.91 ± 2.07%	[57]
	nanoprecipitation						
Homo and sc-PLA/pTHPP@PLGA	nanoparticles in PLGA nanofibers	75:25	200–500/546	-	0.1 to 0.4	-	[58]
	nanoprecipitation and electrospining						
mTHPP-PLGA	nanoparticles	50:50	107.6 ± 6.8	−29.7 ± 2.6	0.07 ± 0.01	-	[59]
	solvent displacement						
mTHPP-CS-PLGA	nanoparticles	50:50	120.1 ± 4.2	+10.3 ± 0.6	0.20 ± 0.01	-	
	solvent displacement						
mTHPP-PLGA-PEG	nanoparticles	NS	93.4 ± 2.3	−19.8 ± 1.7	0.04 ± 0.01	-	
	solvent displacement						
mTHPP-PLGA	nanoparticles	50:50	107.6 ± 6.8	−29.7 ± 2.6	0.07 ± 0.01	-	[60]
	solvent displacement						
mTHPP-CS-PLGA	nanoparticles	50:50	120.1 ± 4.2	+10.3 ± 0.6	0.20 ± 0.01	-	
	solvent displacement						
mTHPP-PLGA-PEG	nanoparticles	NS	93.4 ± 2.3	−19.8 ± 1.7	0.04 ± 0.01	-	
	solvent displacement						
mTHPP-Pd/PLGA	nanoparticles	NS	250	-	<0.1	-	[61]
	emulsion diffusion						

Abbreviations: NS—not specified; PHBV—poly(hydroxybutyrate-co-hydroxyvalerate); PLHNP—polymer-lipid hybrid nanoparticle.

**Table 4:** The physical parameters of Ce6/PLGA nanoparticles.

Name	Type of NP	LA to GA Ratio	Size [nm]		Zeta Potential [mV]	PDI	EE	Ref.
	Preparation Method		DLS	TEM				
Fe_3_O_4_-PLGA-Ce6	nanoparticles	NS	~100	85	−30.1			[67]
	emulsion diffusion							
CPNPs (Ce6/PLGA)	nanoparticles	50:50	232.58 ± 5.73		−18.57 ± 1.875	0.036		[68]
	double-emulsion solvent evaporation							
FTCPNPs (Ce6/PLGA/Fe^3+^-TA)	core-shell NPs	50:50	246.32 ± 5.34		−29.70 ± 1.819	0.086	82.09 ± 2.332% (Ce6)	
	double-emulsion solvent evaporation							
PTFCG	core-shell nanocomposites	NS	~175		−33.4			[69]
	solvent exchange and evaporation							
PTFCG-M	core-shell nanocomposites	NS			+17.3			
	in situ growth							
PTFCG-MH	core-shell nanocomposites	NS	~205	~160	−21.7			
	electrostatic coating							
DOX/PB-Ce6	core-shell structures	NS	164.18–531.17		−38.4		56.18 ± 3.06% (DOX)	[70]
	double emulsion-solvent evaporation						46.16 ± 2.81% (Ce6)	
Lum/Ce6-PLGA	nanoparticles	NS		120				[71]
	O/W single-emulsion							
Ce6-PLGA	nanoparticles	NS		~160				[72]
	self-assembly in aqueous conditions							
PLA/Ce6	nanoparticles	NS	183 ± 30			0.046	10.4 ± 1.4%	[73]
	salting-out or emulsification-diffusion							

Abbreviations: NS—not specified; PTFCG—Ce6, FeCl_3_ and PLGA hybrid nanoparticles; PTFCG-M—Ce6, FeCl_3_, PLGA and poly(allylamine hydrochloride) hybrid nanoparticles; PTFCG-MH—Ce6, FeCl_3_, PLGA, poly(allylamine hydrochloride) and hyaluronic acid hybrid nanoparticles.

**Table 5:** The physical parameters of PLGA nanoparticles described in Section 2.4.

Name	Type of NP	LA to GA Ratio	Size [nm]		Zeta Potential [mV]	PDI	EE	Ref.
	Preparation Method		DLS	TEM				
PLA/TPP	nanoparticles	NS	210 ± 11	-	-	0.323	87.2 ± 1.0%	[73]
	salting-out							
PLA/TCPP	nanoparticles	NS	194 ± 12	-	-	0.219	20.9 ± 0.5%	
	salting-out							
PLA/Pheo	nanoparticles	NS	233 ± 10	-	-	0.256	14.4 ± 0.7%	
	salting-out							
PLGA-VP	nanoparticles	50:50	241 ± 4–252 ± 4	−20 ±1 to −23.3 ± 0.1	0.03 ± 0.01–0.09 ± 0.02	-	4.5–39.6 µM	[75]
	single emulsion solvent evaporation							
NiTPP/PLGA	nanoparticles	50:50	322.9 ± 9.7	-	−14.7 ± 1.7	0.172	24.1 ± 0.9%	[77]
	single emulsion solvent evaporation							
CoTPP/PLGA	nanoparticles	50:50	344.5 ± 15.6	-	−10.7 ± 2.3	0.191	79.7 ± 2.2%	
	single emulsion solvent evaporation							
MnClTPP/PLGA	nanoparticles	50:50	205.2 ± 10.2	-	+18.1 ± 1.6 mV	0.140	79.9 ± 1.8%	
	single emulsion solvent evaporation							
PLGA-Arg	nanoparticles	50:50	121.1 ± 11.19	-	+6.02 ± 2.11	-	-	[78]
	emulsion solvent evaporation							
PLGA-Arg-Hematin	nanoparticles	50:50	127.5 ± 9.93	-	−15.19 ± 2.43	-	-	
	emulsion solvent evaporation							
PLGA + VP + CisPt	nanoparticles	50:50	193 ± 6	-	−9 ± 1	0.16 ± 0.01	92 ± 1% (CisPt) 97 ± 3% (VP)	[76]
	double-emulsion solvent evaporation							
PLGA-PEG + VP + CisPt	nanoparticles	50:50	187 ± 5	-	−4 ± 1	0.12 ± 0.01	88 ± 1% (CisPt) 92 ± 1% (VP)	
	double-emulsion solvent evaporation							
PLGA-FA + VP + CisPt	nanoparticles	50:50	200 ± 7	-	−15 ± 2	0.20 ± 0.02	90 ± 2% (CisPt) 95 ± 3% (VP)	
	double-emulsion solvent evaporation							
PLGA-FA + VP	nanoparticles	50:50	197 ± 7	-	−16 ± 2	0.22 ± 0.02	96 ± 3 (VP)	
	double-emulsion solvent evaporation							
PLGA-FA + CisPt	nanoparticles	50:50	194 ± 6	-	−16 ± 2	0.25 ± 0.02	92 ± 2 (CisPt)	
	double-emulsion solvent evaporation							
PLGA-FA	nanoparticles	50:50	189 ± 5	-	−17 ± 3	0.10 ± 0.01	-	
	double-emulsion solvent evaporation							
TMPyP-PLGA	nanoparticles	50:50	118 ± 5–133 ± 3	-	−26.7 ± 3.0 to −21.6 ± 1.0	0.17 ± 0.04 to 0.18 ± 0.03	55.8 ± 1.1 to 92.5 ± 35%	[79]
	evaporation method							
MP/TMPyP	nanoparticles	75:25	117	-	-	0.066	59.7%	[81]
	double-emulsion solvent evaporation							
TCPP/Iso/PEG-b-PLGA	nanoparticles	50:50	87–108	-	-	0.2–0.4	-	[87]
	anti-solvent precipitation process							
PLGA/OVA	microparticles	50:50	0.4–1.3 µm	-	−20 to–12	-	69%	[88]
	double-emulsion solvent evaporation							
PLGA/OVA/TPCS2a	microparticles	50:50	0.4–1.3 µm	-	−20 to–12	-	58%	
	double-emulsion solvent evaporation							
PLGA-mTHPC-CP	nanoparticles	NS	124.1 ± 2.8	-	−52.8 ± 1.5	0.03 ± 0.01	-	[89]
	double-emulsion solvent evaporation							
PLGA-mTHPC-F127	nanoparticles	NS	115.4 ± 0.8	-	−46,5 ± 4.5	0.09 ± 0.01	-	
	double-emulsion solvent evaporation							
PLGA-(Chl)-QD	nanoparticles	75:25	169–220	-	−30	-	20% (Chl) 90% (QDs)	[92]
	nanoprecipitation method							
PLGA-bFGF	nanoparticles	50:50	415 ± 25.6	-	−9.86 ± 1.5	-	-	[93]
	double-emulsion solvent evaporation							
TBPP/PLGA	nanoparticles	75:25	~100	-	−39	0.1	-	[96]
	dialysis method							
TTA-UC PLGA	nanoparticles	50:50	200 ± 50	-	−31 ± 5	0.4	24.4% (PtOEP) 39.6% (DPA)	[98]
PLGA/PMnC/GOx	nanoparticles	50:50	278.3 ± 40.96	-	−26.7 ± 3.29	-	95.83 ± 1.35% (PMnC) 28.80 ± 1.96% (GOx)	[99]
	double-emulsion solvent evaporation							

Abbreviations: NS—not specified.

## Data Availability

No new data were created or analysed in this study.

## Abbreviations

The following abbreviations are used in this manuscript:

[EP+D]	epidermis plus dermis
5-ALA	5-aminolevulinic acid
AFM	Atomic Force Microscope
ALA	alanine
ART	artemisinin
ATP	adenosine triphosphate
AuNPs	gold nanoparticles
BBD	Box–Behnken design
bFGF	fibroblast growth factor
BNCT	boron neutron capture therapy
BRET	bioluminescence resonance energy transfer
BSA	bovine serum albumin
CAM	chick embryo chorioallantoic membrane
CDS	chondroitin sulphate
Ce6	Chlorin e6
Chl	chlorophyllin copper complex
CisPt	Cisplatin
CLP	cecal ligation and puncture
CMC	critical micelle concentration
CS	chitosan
DCC	*N,N’*-dicyclohexylcarbodiimide
DCM	dichloromethane
DDS	drug-delivery system
DHR123	dihydrorhodamine 123
DLC	Drug-Loading Content
DLS	dynamic light scattering
DMAP	4-dimethylaminopyridine
DMEM	Dulbecco’s Modified Eagle Medium
DMSO	dimethyl sulfoxide
DOX	doxycycline
DPA	9,10-diphenylantracene
DPPC	1,2-dipalmitoyl-*sn*-glycero-3-phosphocholine
DPPG	1,2-dipalmitoyl-*sn*-glycero-3-phosphoglycerol
DSC	differential scanning calorimetry
DSPE-PEG-COOH	(1,2-distearoyl-*sn*-glycero-3-phosphoethanolamine-*N*-[carboxyl(poly-etylene glycol)-200]
DVDMS	sinoporphyrin sodium
EC_50_	half maximal effective concentration
EDC	1-ethyl-3-(3-dimethylaminopropyl)carbodiimide
EDX	Energy-dispersive X-ray
EE	Encapsulation Efficiency
EPR	Enhanced permeability and retention
EtOAc	ethyl acetate
Eudragit E^®^	poly(butyl methacrylate-co-(2-dimethylaminoethyl) methacrylate-co-methylmethacrylate
FA	folic acid
FBS	fetal bovine serum
FDA	Food and Drug Administration
FRET	Förster resonance energy transfer
FTIR	Fourier-transform infrared spectroscopy
GCTB	giant cell tumours of bone
GOx	glucose oxidase
GSH	glutathione
GT	gelatin
HAp	hydroxyapatite
HA	hyaluronic acid
HA-b-PLGA	hyaluronic acid-block-poly(D,L-lactide-co-glycolide)
HAT	hyaluronic acid tyramine
HBA	carboxyl phenylhydrazine
HEPES	4-(2-hydroxyethyl)-1-piperazineethanesulfonic acid
HepG2	human hepatoma
HPLC-DAD	High-Performance Liquid Chromatography with Diode-Array Detection
IC_50_	half maximal inhibitory concentration
ICG	indocyanine green
ICP-OES	Inductively Coupled Plasma Optical Emission Spectrometry
Iso	isolienisinine
KB	human nasopharyngeal epidermal carcinoma
LA-ICP-MS	Laser Ablation Inductively Coupled Plasma Mass Spectrometry
LDH	lactate dehydrogenase
LPS	lipopolysaccharide
MDR	multidrug-resistant
mMSCs	mesenchymal stem cells
MN	microneedle
MOF	metal–organic framework
MPO	myeloperoxidase
MPs	*meso*-tetraphenylporphyrins
MR/PA	magnetic resonance/photoacoustic
MRI	magnetic resonance imaging
mTHPC	5,10,15,20-tetrakis(pentafluorophenyl)porphyrin
mTHPP	5,10,15,20-tetrakis(3-hydroxyphenyl)porphin
mTHPP-Pd	[5,10,15,20-tetrakis(3-hydroxyphenyl)porphyrinato]palladium(II)
MTT	3-(4,5-dimethylthiazolyl-2)-2,5-diphenyltetrazolium bromide
NHS	N-hydroxysuccinimide
NIR	near-infrared
NMR	Nuclear Magnetic Resonance spectroscopy
NPs	nanoparticles
NR	Nile Red
O/W	oil in water
OA	oleic acid
OCDs	osteochondral defects
OVA	ovalbumin
PACT	Photodynamic Antimicrobial Chemotherapy
Pba, Pheo-a	pheophorbide a
PBG	porphobilinogen
PBS	phosphate-buffered saline
PC3	prostate cancer cells
PCL	polycaprolactone
PDD	Photodynamic Diagnostics
PDI	polydispersity index
PDT	photodynamic therapy
PEG	polyethylene glycol
PET	positron emission tomography
PHBV	poly(3-hydroxybutyrate-co-3-hydroxyvalerate)
PLA	polylactic acid
PLGA	poly(lactic-co-glycolic acid)
PPIX	protoporphyrin IX
PS	photosensitizer
pTHPP	5,10,15,20-tetrakis(4-hydroxyphenyl)porphin
PtOEP	platinum octaethylporphyrin
PTT	photothermal therapy
PVA	poly(vinyl alcohol)
QDs	quantum dots
ROS	reactive oxygen species
RT-PCR	Real-Time Reverse-Transcription Polymerase Chain Reaction
SC	stratum corneum
SCC	squamous cell carcinoma
SDT	Sonodynamic Therapy
SEM	Scanning Electron Microscope
SOSG	Singlet Oxygen Sensor Green
TA	tannic acid
TCPP	5,10,15,20-tetrakis(4-carboxyphenyl)porphyrin
TEA	triethylamine
TEM	Transmission Electron Microscopy
TH	trehalose
THF	tetrahydrofuran
TMPyP	meso-tetrakis(1-methylpyridinium-4-yl)
TPP	5,10,15,20-tetraphenylporphyrin
TRAP	Telomerase Repeated Amplification Protocol
TTA-UC	Triplet-Triplet Annihilation Upconversion
Tyr	tyramine
UCNPs	upconversion nanoparticles
US	ultrasound
UV-Vis	Ultraviolet-Visible spectroscopy
VP	Verteporfin, Visudyne^®^
VSV	vesicular stomatitis virus
w_1_/o/w_2_	water-in-oil-in-water
XRD	X-Ray Diffraction
XRPD	X-Ray Powder Diffraction

## References

[1] Niculescu A.G., Grumezescu A.M. (2021). Photodynamic Therapy—An up-to-Date Review. Appl. Sci..

[2] Correia J.H., Rodrigues J.A., Pimenta S., Dong T., Yang Z. (2021). Photodynamic Therapy Review: Principles, Photosensitizers, Applications, and Future Directions. Pharmaceutics.

[3] Zhou Z., Song J., Nie L., Chen X. (2016). Reactive Oxygen Species Generating Systems Meeting Challenges of Photodynamic Cancer Therapy. Chem. Soc. Rev..

[4] Kwiatkowski S., Knap B., Przystupski D., Saczko J., Kędzierska E., Knap-Czop K., Kotlińska J., Michel O., Kotowski K., Kulbacka J. (2018). Photodynamic Therapy—Mechanisms, Photosensitizers and Combinations. Biomed. Pharmacother..

[5] Sun B., Bte Rahmat J.N., Zhang Y. (2022). Advanced Techniques for Performing Photodynamic Therapy in Deep-Seated Tissues. Biomaterials.

[6] Hu Z. (2014). Photodynamic Therapy as an Emerging Treatment Modality for Cancer and Non-Cancer Diseases. J. Anal. Bioanal. Tech..

[7] Lan M., Zhao S., Liu W., Lee C.S., Zhang W., Wang P. (2019). Photosensitizers for Photodynamic Therapy. Adv. Healthc. Mater..

[8] Kou J., Dou D., Yang L. (2017). Porphyrin Photosensitizers in Photodynamic Therapy and Its Applications. Oncotarget.

[9] Zhao X., Liu J., Fan J., Chao H., Peng X. (2021). Recent Progress in Photosensitizers for Overcoming the Challenges of Photodynamic Therapy: From Molecular Design to Application. Chem. Soc. Rev..

[10] Glowacka-Sobotta A., Czarczynska-Goslinska B., Ziental D., Wysocki M., Michalak M., Güzel E., Sobotta L. (2024). Versatile Porphyrin Arrangements for Photodynamic Therapy—A Review. Nanomaterials.

[11] Liechty W.B., Kryscio D.R., Slaughter B.V., Peppas N.A. (2010). Polymers for Drug Delivery Systems. Annu. Rev. Chem. Biomol. Eng..

[12] Joralemon M.J., McRae S., Emrick T. (2010). PEGylated Polymers for Medicine: From Conjugation to Self-Assembled Systems. Chem. Commun..

[13] Danhier F., Ansorena E., Silva J.M., Coco R., Le Breton A., Préat V. (2012). PLGA-Based Nanoparticles: An Overview of Biomedical Applications. J. Control. Release.

[14] De las Heras Alarcón C., Pennadam S., Alexander C. (2005). Stimuli Responsive Polymers for Biomedical Applications. Chem. Soc. Rev..

[15] Avramović N., Mandić B., Savić-Radojević A., Simić T. (2020). Polymeric Nanocarriers of Drug Delivery Systems in Cancer Therapy. Pharmaceutics.

[16] Salari N., Faraji F., Torghabeh F.M., Faraji F., Mansouri K., Abam F., Shohaimi S., Akbari H., Mohammadi M. (2022). Polymer-Based Drug Delivery Systems for Anticancer Drugs: A Systematic Review. Cancer Treat. Res. Commun..

[17] Vilar G., Tulla-Puche J., Albericio F. (2012). Polymers and Drug Delivery Systems. Curr. Drug Deliv..

[18] Jain V., Jain S., Mahajan S.C. (2015). Nanomedicines Based Drug Delivery Systems for Anti-Cancer Targeting and Treatment. Curr. Drug Deliv..

[19] Makadia H.K., Siegel S.J. (2011). Poly Lactic-Co-Glycolic Acid (PLGA) as Biodegradable Controlled Drug Delivery Carrier. Polymers.

[20] Kapoor D.N., Bhatia A., Kaur R., Sharma R., Kaur G., Dhawan S. (2015). PLGA: A Unique Polymer for Drug Delivery. Ther. Deliv..

[21] Martins C., Sousa F., Araújo F., Sarmento B. (2018). Functionalizing PLGA and PLGA Derivatives for Drug Delivery and Tissue Regeneration Applications. Adv. Healthc. Mater..

[22] Xu Y., Kim C.S., Saylor D.M., Koo D. (2017). Polymer Degradation and Drug Delivery in PLGA-Based Drug–Polymer Applications: A Review of Experiments and Theories. J. Biomed. Mater. Res. Part B Appl. Biomater..

[23] Mehraban N., Musich P.R., Freeman H.S. (2019). Synthesis and Encapsulation of a New Zinc Phthalocyanine Photosensitizer into Polymeric Nanoparticles to Enhance Cell Uptake and Phototoxicity. Appl. Sci..

[24] de Toledo M.C.M.C., da Silva Abreu A., Carvalho J.A., Ambrósio J.A.R., da Silva Godoy D., Pinto B.C.D.S., Junior M.B., Simioni A.R. (2020). Zinc Phthalocyanine Tetrasulfonate-Loaded Polyelectrolytic PLGA Nanoparticles for Photodynamic Therapy Applications. Photodiagn. Photodyn. Ther..

[25] Fadel M., Kassab K., Abdel Fadeel D. (2010). Zinc Phthalocyanine-Loaded PLGA Biodegradable Nanoparticles for Photodynamic Therapy in Tumor-Bearing Mice. Lasers Med. Sci..

[26] Borzęcka W., Domiński A., Kowalczuk M. (2021). Recent Progress in Phthalocyanine-Polymeric Nanoparticle Delivery Systems for Cancer Photodynamic Therapy. Nanomaterials.

[27] Rak J., Pouckova P., Benes J., Vetvicka D. (2019). Drug Delivery Systems for Phthalocyanines for Photodynamic Therapy. Anticancer Res..

[28] Hussain Z., Qi Q., Zhu J., Anderson K.E., Ma X. (2023). Protoporphyrin IX-Induced Phototoxicity: Mechanisms and Therapeutics. Pharmacol. Ther..

[29] Sitte E., Senge M.O. (2020). The Red Color of Life Transformed—Synthetic Advances and Emerging Applications of Protoporphyrin IX in Chemical Biology. Eur. J. Org. Chem..

[30] Nyman E.S., Hynninen P.H. (2004). Research Advances in the Use of Tetrapyrrolic Photosensitizers for Photodynamic Therapy. J. Photochem. Photobiol. B Biol..

[31] Peng Q., Berg K., Moan J., Kongshaug M., Nesland J.M. (1997). 5-Aminolevulinic Acid-Based Photodynamic Therapy: Principles and Experimental Research. Photochem. Photobiol..

[32] Chen J., Fan T., Xie Z., Zeng Q., Xue P., Zheng T., Chen Y., Luo X., Zhang H. (2020). Advances in Nanomaterials for Photodynamic Therapy Applications: Status and Challenges. Biomaterials.

[33] Da Silva C.L., Del Ciampo J.O., Rossetti F.C., Bentley M.V.L.B., Pierre M.B.R. (2013). Improved in Vitro and in Vivo Cutaneous Delivery of Protoporphyrin IX from PLGA-Based Nanoparticles. Photochem. Photobiol..

[34] Da Silva C.L., Del Ciampo J.O., Cristina Rossetti F., Badra Bentley M.V.L., Riemma Pierre M.B. (2013). PLGA Nanoparticles as Delivery Systems for Protoporphyrin Ix in Topical Pdt: Cutaneous Penetration of Photosensitizer Observed by Fluorescence Microscopy. J. Nanosci. Nanotechnol..

[35] da Silva D.B., da Silva C.L., Davanzo N.N., da Silva Souza R., Correa R.J., Tedesco A.C., Riemma Pierre M.B. (2021). Protoporphyrin IX (PpIX) Loaded PLGA Nanoparticles for Topical Photodynamic Therapy of Melanoma Cells. Photodiagn. Photodyn. Ther..

[36] Izquierdo N., Gamez E., Alejo T., Mendoza G., Arruebo M. (2024). Antimicrobial Photodynamic Therapy Using Encapsulated Protoporphyrin IX for the Treatment of Bacterial Pathogens. Materials.

[37] Wang X., Wang J., Li J., Huang H., Sun X., Lv Y. (2018). Development and Evaluation of Hyaluronic Acid-Based Polymeric Micelles for Targeted Delivery of Photosensitizer for Photodynamic Therapy in Vitro. J. Drug Deliv. Sci. Technol..

[38] Wan G.Y., Liu Y., Chen B.W., Liu Y.Y., Wang Y.S., Zhang N. (2016). Recent Advances of Sonodynamic Therapy in Cancer Treatment. Cancer Biol. Med..

[39] Yan P., Liu L.H., Wang P. (2020). Sonodynamic Therapy (SDT) for Cancer Treatment: Advanced Sensitizers by Ultrasound Activation to Injury Tumor. ACS Appl. Bio Mater..

[40] Dong H.-Q., Fu X.-F., Wang M.-Y., Zhu J. (2023). Research Progress on Reactive Oxygen Species Production Mechanisms in Tumor Sonodynamic Therapy. World J. Clin. Cases.

[41] He W., Li C., Zhao S., Li Z., Wu J., Li J., Zhou H., Yang Y., Xu Y., Xia H. (2024). Integrating Coaxial Electrospinning and 3D Printing Technologies for the Development of Biphasic Porous Scaffolds Enabling Spatiotemporal Control in Tumor Ablation and Osteochondral Regeneration. Bioact. Mater..

[42] Prieto M., Rwei A.Y., Alejo T., Wei T., Lopez-Franco M.T., Mendoza G., Sebastian V., Kohane D.S., Arruebo M. (2017). Light-Emitting Photon-Upconversion Nanoparticles in the Generation of Transdermal Reactive-Oxygen Species. ACS Appl. Mater. Interfaces.

[43] Zou X., Yao M., Ma L., Hossu M., Han X., Juzenas P., Chen W. (2014). X-Ray-Induced Nanoparticle-Based Photodynamic Therapy of Cancer. Nanomedicine.

[44] Dinakaran D., Sengupta J., Pink D., Raturi A., Chen H., Usmani N., Kumar P., Lewis J.D., Narain R., Moore R.B. (2020). PEG-PLGA Nanospheres Loaded with Nanoscintillators and Photosensitizers for Radiation-Activated Photodynamic Therapy. Acta Biomater..

[45] Azad A.K., Lilge L., Usmani N.H., Lewis J.D., Cole H.D., Cameron C.G., McFarland S.A., Dinakaran D., Moore R.B. (2023). High Quantum Efficiency Ruthenium Coordination Complex Photosensitizer for Improved Radiation-Activated Photodynamic Therapy. Front. Oncol..

[46] Shi L., Wang X., Zhao F., Luan H., Tu Q., Huang Z., Wang H., Wang H. (2013). In Vitro Evaluation of 5-Aminolevulinic Acid (ALA) Loaded PLGA Nanoparticles. Int. J. Nanomed..

[47] Wang X., Shi L., Tu Q., Wang H., Zhang H., Wang P., Zhang L., Huang Z., Zhao F., Luan H. (2015). Treating Cutaneous Squamous Cell Carcinoma Using 5-Aminolevulinic Acid Polylactic-Co-Glycolic Acid Nanoparticle-Mediated Photodynamic Therapy in a Mouse Model. Int. J. Nanomed..

[48] Wang L., Hu Y., Hao Y., Li L., Zheng C., Zhao H., Niu M., Yin Y., Zhang Z., Zhang Y. (2018). Tumor-Targeting Core-Shell Structured Nanoparticles for Drug Procedural Controlled Release and Cancer Sonodynamic Combined Therapy. J. Control. Release.

[49] Li Z., Song Y., Luo Q., Liu Z., Man Y., Liu J., Lu Y., Zheng L. (2024). Carrier Cascade Target Delivery of 5-Aminolevulinic Acid Nanoplatform to Enhance Antitumor Efficiency of Photodynamic Therapy against Lung Cancer. J. Photochem. Photobiol. B Biol..

[50] Konan Y.N., Berton M., Gurny R., Allémann E. (2003). Enhanced Photodynamic Activity of Meso-Tetra(4-Hydroxyphenyl)Porphyrin by Incorporation into Sub-200 Nm Nanoparticles. Eur. J. Pharm. Sci..

[51] Konan Y.N., Cerny R., Favet J., Berton M., Gurny R., Allémann E. (2003). Preparation and Characterization of Sterile Sub-200 Nm Meso-Tetra(4-Hydroxylphenyl)Porphyrin-Loaded Nanoparticles for Photodynamic Therapy. Eur. J. Pharm. Biopharm..

[52] Konan Y.N., Chevallier J., Gurny R., Allémann E. (2003). Encapsulation of P-THPP into Nanoparticles: Cellular Uptake, Subcellular Localization and Effect of Serum on Photodynamic Activity. Photochem. Photobiol..

[53] Vargas A., Pegaz B., Debefve E., Konan-Kouakou Y., Lange N., Ballini J.P., Van Den Bergh H., Gurny R., Delie F. (2004). Improved Photodynamic Activity of Porphyrin Loaded into Nanoparticles: An in Vivo Evaluation Using Chick Embryos. Int. J. Pharm..

[54] Vargas A., Lange N., Arvinte T., Cerny R., Gurny R., Delie F. (2009). Toward the Understanding of the Photodynamic Activity of M-THPP Encapsulated in PLGA Nanoparticles: Correlation between Nanoparticle Properties and in Vivo Activity. J. Drug Target..

[55] Grünebaum J., Söbbing J., Mulac D., Langer K. (2015). Nanoparticulate Carriers for Photodynamic Therapy of Cholangiocarcinoma: In Vitro Comparison of Various Polymer-Based Nanoparticles. Int. J. Pharm..

[56] Pramual S., Lirdprapamongkol K., Svasti J., Bergkvist M., Jouan-Hureaux V., Arnoux P., Frochot C., Barberi-Heyob M., Niamsiri N. (2017). Polymer-Lipid-PEG Hybrid Nanoparticles as Photosensitizer Carrier for Photodynamic Therapy. J. Photochem. Photobiol. B Biol..

[57] Pramual S., Lirdprapamongkol K., Jouan-Hureaux V., Barberi-Heyob M., Frochot C., Svasti J., Niamsiri N. (2020). Overcoming the Diverse Mechanisms of Multidrug Resistance in Lung Cancer Cells by Photodynamic Therapy Using PTHPP-Loaded PLGA-Lipid Hybrid Nanoparticles. Eur. J. Pharm. Biopharm..

[58] Forouharshad M., Ajalloueian F. (2022). Tunable Self-Assembled Stereocomplexed-Polylactic Acid Nanoparticles as a Drug Carrier. Polym. Adv. Technol..

[59] Mahlert L., Anderski J., Mulac D., Langer K. (2019). The Impact of Gastrointestinal Mucus on Nanoparticle Penetration—In Vitro Evaluation of Mucus-Penetrating Nanoparticles for Photodynamic Therapy. Eur. J. Pharm. Sci..

[60] Anderski J., Mahlert L., Mulac D., Langer K. (2018). Mucus-Penetrating Nanoparticles: Promising Drug Delivery Systems for the Photodynamic Therapy of Intestinal Cancer. Eur. J. Pharm. Biopharm..

[61] Niehoff A.C., Moosmann A., Söbbing J., Wiehe A., Mulac D., Wehe C.A., Reifschneider O., Blaske F., Wagner S., Sperling M. (2014). A Palladium Label to Monitor Nanoparticle-Assisted Drug Delivery of a Photosensitizer into Tumor Spheroids by Elemental Bioimaging. Metallomics.

[62] Hak A., Ali M.S., Sankaranarayanan S.A., Shinde V.R., Rengan A.K. (2023). Chlorin E6: A Promising Photosensitizer in Photo-Based Cancer Nanomedicine. ACS Appl. Bio Mater..

[63] Liao S., Cai M., Zhu R., Fu T., Du Y., Kong J., Zhang Y., Qu C., Dong X., Ni J. (2023). Antitumor Effect of Photodynamic Therapy/Sonodynamic Therapy/Sono-Photodynamic Therapy of Chlorin E6 and Other Applications. Mol. Pharm..

[64] Michalak M., Szymczyk J., Pawska A., Wysocki M., Janiak D., Ziental D., Ptaszek M., Güzel E., Sobotta L. (2025). Chlorin Activity Enhancers for Photodynamic Therapy. Molecules.

[65] Park J.H., Moon Y.H., Bang I.S., Kim Y.C., Kim S.A., Ahn S.G., Yoon J.H. (2010). Antimicrobial Effect of Photodynamic Therapy Using a Highly Pure Chlorin E6. Lasers Med. Sci..

[66] Lee D.J., Park G.Y., Oh K.T., Oh N.M., Kwag D.S., Youn Y.S., Oh Y.T., Park J.W., Lee E.S. (2012). Multifunctional Poly (Lactide-Co-Glycolide) Nanoparticles for Luminescence/Magnetic Resonance Imaging and Photodynamic Therapy. Int. J. Pharm..

[67] Chen Q., Ma X., Xie L., Chen W., Xu Z., Song E., Zhu X., Song Y. (2021). Iron-Based Nanoparticles for MR Imaging-Guided Ferroptosis in Combination with Photodynamic Therapy to Enhance Cancer Treatment. Nanoscale.

[68] Huang T., Xu X., Cheng C., Wang J., Yang L. (2023). Cooperative Phototherapy Based on Bimodal Imaging Guidance for the Treatment of Uveal Melanoma. J. Nanobiotechnol..

[69] Liu P., Zhou Y., Shi X., Yuan Y., Peng Y., Hua S., Luo Q., Ding J., Li Y., Zhou W. (2021). A Cyclic Nano-Reactor Achieving Enhanced Photodynamic Tumor Therapy by Reversing Multiple Resistances. J. Nanobiotechnol..

[70] Liang J., Jin X., Chen B., Hu J., Huang Q., Wan J., Hu Z., Wang B. (2020). Doxorubicin-Loaded PH-Responsive Nanoparticles Coated with Chlorin E6 for Drug Delivery and Synergetic Chemo-Photodynamic Therapy. Nanotechnology.

[71] Lv X., Min J., Huang J., Wang H., Wei S., Huang C., Dai J., Chen Z., Zhou H., Xu Y. (2024). Simultaneously Controlling Inflammation and Infection by Smart Nanomedicine Responding to the Inflammatory Microenvironment. Adv. Sci..

[72] Son J., Lee D., Yoo J., Park C., Koo H. (2020). A Comparative Study of the Effect of Drug Hydrophobicity on Nanoparticle Drug Delivery in Vivo Using Two Photosensitizers. Nanomed. Nanotechnol. Biol. Med..

[73] Pegaz B., Debefve E., Borle F., Ballini J.P., van den Bergh H., Kouakou-Konan Y.N. (2005). Encapsulation of Porphyrins and Chlorins in Biodegradable Nanoparticles: The Effect of Dye Lipophilicity on the Extravasation and the Photothrombic Activity. A Comparative Study. J. Photochem. Photobiol. B Biol..

[74] Oba T. (2007). Photosensitizer Nanoparticles for Photodynamic Therapy. Curr. Bioact. Compd..

[75] Clement S., Chen W., Deng W., Goldys E.M. (2018). X-Ray Radiation-Induced and Targeted Photodynamic Therapy with Folic Acid-Conjugated Biodegradable Nanoconstructs. Int. J. Nanomed..

[76] Bazylińska U., Kulbacka J., Chodaczek G. (2019). Nanoemulsion Structural Design in Co-Encapsulation of Hybrid Multifunctional Agents: Influence of the Smart Plga Polymers on the Nanosystem-Enhanced Delivery and Electro-Photodynamic Treatment. Pharmaceutics.

[77] Mollaeva M.R., Yabbarov N., Sokol M., Chirkina M., Mollaev M.D., Zabolotskii A., Seregina I., Bolshov M., Kaplun A., Nikolskaya E. (2021). Optimization, Characterization and Pharmacokinetic Study of Meso-tetraphenylporphyrin Metal Complex-loaded Plga Nanoparticles. Int. J. Mol. Sci..

[78] Amin M.L., Kim D., Kim S.J. (2016). Development of Hematin Conjugated PLGA Nanoparticle for Selective Cancer Targeting. Eur. J. Pharm. Sci..

[79] González-Delgado J.A., Castro P.M., Machado A., Araújo F., Rodrigues F., Korsak B., Ferreira M., Tomé J.P.C., Sarmento B. (2016). Hydrogels Containing Porphyrin-Loaded Nanoparticles for Topical Photodynamic Applications. Int. J. Pharm..

[80] Gomes M.C., Woranovicz-Barreira S.M., Faustino M.A.F., Fernandes R., Neves M.G.P.M.S., Tomé A.C., Gomes N.C.M., Almeida A., Cavaleiro J.A.S., Cunha Â. (2011). Photodynamic Inactivation of Penicillium Chrysogenum Conidia by Cationic Porphyrins. Photochem. Photobiol. Sci..

[81] Chi Y., Zheng Y., Pan X., Huang Y., Kang Y., Zhong W., Xu K. (2024). Enzyme-Mediated Fabrication of Nanocomposite Hydrogel Microneedles for Tunable Mechanical Strength and Controllable Transdermal Efficiency. Acta Biomater..

[82] Ito F., Yamada H., Kanamura K., Kawakami H. (2019). Preparation of Biodegradable Polymer Nanospheres Containing Manganese Porphyrin (Mn-Porphyrin). J. Inorg. Organomet. Polym. Mater..

[83] Ohse T., Kawakami H., Morita A., Nagaoka S. (1999). Mn-Porphyrin Derivatives as an Antioxidant for Medical Devices. J. Biomater. Sci. Polym. Ed..

[84] Laster B.H., Isaacson C., Perets E., Msamra M., Priel E., Kalef-Ezra J., Kost J. (2015). Keeping Those Telomeres Short! An Innovative Intratumoral Long-Term Drug Delivery System. J. Cancer Res. Clin. Oncol..

[85] Hu Z., Pan Y., Wang J., Chen J., Li J., Ren L. (2009). Meso-Tetra (Carboxyphenyl) Porphyrin (TCPP) Nanoparticles Were Internalized by SW480 Cells by a Clathrin-Mediated Endocytosis Pathway to Induce High Photocytotoxicity. Biomed. Pharmacother..

[86] Gong X., Milic T., Xu C., Batteas J.D., Drain C.M. (2002). Preparation and Characterization of Porphyrin Nanoparticles. J. Am. Chem. Soc..

[87] Zhang C., Wang X., Wang J., Qiu Y., Qi Z., Song D., Wang M. (2021). Tcpp-Isoliensinine Nanoparticles for Mild-Temperature Photothermal Therapy. Int. J. Nanomed..

[88] Bruno C., Waeckerle-Men Y., Håkerud M., Kündig T.M., Gander B., Johansen P. (2015). Photosensitizer and Light Pave the Way for Cytosolic Targeting and Generation of Cytosolic CD8 T Cells Using PLGA Vaccine Particles. J. Immunol..

[89] Elberskirch L., Le Harzic R., Scheglmann D., Wieland G., Wiehe A., Mathieu-Gaedke M., Golf H.R.A., von Briesen H., Wagner S. (2022). A HET-CAM Based Vascularized Intestine Tumor Model as a Screening Platform for Nano-Formulated Photosensitizers. Eur. J. Pharm. Sci..

[90] Geier G.R., Lindsey J.S. (2004). Effects of Aldehyde or Dipyrromethane Substituents on the Reaction Course Leading to Meso-Substituted Porphyrins. Tetrahedron.

[91] Xu Q., Boylan N.J., Cai S., Miao B., Patel H., Hanes J. (2013). Scalable Method to Produce Biodegradable Nanoparticles That Rapidly Penetrate Human Mucus. J. Control. Release.

[92] Galliani M., Signore G. (2019). Poly(Lactide-Co-Glycolide) Nanoparticles Co-Loaded with Chlorophyllin and Quantum Dots as Photodynamic Therapy Agents. Chempluschem.

[93] Mai B., Jia M., Liu S., Sheng Z., Li M., Gao Y., Wang X., Liu Q., Wang P. (2020). Smart Hydrogel-Based DVDMS/BFGF Nanohybrids for Antibacterial Phototherapy with Multiple Damaging Sites and Accelerated Wound Healing. ACS Appl. Mater. Interfaces.

[94] Park J.S., Yang H.N., Woo D.G., Jeon S.Y., Park K.H. (2013). Multilineage Differentiation of Human-Derived Dermal Fibroblasts Transfected with Genes Coated on PLGA Nanoparticles plus Growth Factors. Biomaterials.

[95] Presley K.F., Reinsch B.M., Cybyk D.B., Ly J.T., Schweller R.M., Dalton M.J., Lannutti J.J., Grusenmeyer T.A. (2021). Oxygen Sensing Performance of Biodegradable Electrospun Nanofibers: Influence of Fiber Composition and Core-Shell Geometry. Sens. Actuators B Chem..

[96] Shi Y., Li J., Zhang Z., Duan D., Zhang Z., Liu H., Liu T., Liu Z. (2018). Tracing Boron with Fluorescence and Positron Emission Tomography Imaging of Boronated Porphyrin Nanocomplex for Imaging-Guided Boron Neutron Capture Therapy. ACS Appl. Mater. Interfaces.

[97] Chen M., Gao S., Dong M., Song J., Yang C., Howard K.A., Kjems J., Besenbacher F. (2012). Chitosan/SiRNA Nanoparticles Encapsulated in PLGA Nanofibers for SiRNA Delivery. ACS Nano.

[98] Vepris O., Eich C., Feng Y., Fuentes G., Zhang H., Kaijzel E.L., Cruz L.J. (2022). Optically Coupled PtOEP and DPA Molecules Encapsulated into PLGA-Nanoparticles for Cancer Bioimaging. Biomedicines.

[99] Wang J., Huang J., Zhou W., Zhao J., Peng Q., Zhang L., Wang Z., Li P., Li R. (2021). Hypoxia Modulation by Dual-Drug Nanoparticles for Enhanced Synergistic Sonodynamic and Starvation Therapy. J. Nanobiotechnology.

[100] Chenthamara D., Subramaniam S., Ramakrishnan S.G., Krishnaswamy S., Essa M.M., Lin F.H., Qoronfleh M.W. (2019). Therapeutic Efficacy of Nanoparticles and Routes of Administration. Biomater. Res..

[101] Ricci-Júnior E., Marchetti J.M. (2006). Preparation, Characterization, Photocytotoxicity Assay of PLGA Nanoparticles Containing Zinc (II) Phthalocyanine for Photodynamic Therapy Use. J. Microencapsul..

[102] Hung H.I., Klein O.J., Peterson S.W., Rokosh S.R., Osseiran S., Nowell N.H., Evans C.L. (2016). PLGA Nanoparticle Encapsulation Reduces Toxicity While Retaining the Therapeutic Efficacy of EtNBS-PDT in Vitro. Sci. Rep..

[103] Liu Y., Yang G., Jin S., Xu L., Zhao C.X. (2020). Development of High-Drug-Loading Nanoparticles. Chempluschem.

